# Dynamics of Aspen Roots Colonization by Pseudomonads Reveals Strain-Specific and Mycorrhizal-Specific Patterns of Biofilm Formation

**DOI:** 10.3389/fmicb.2018.00853

**Published:** 2018-05-03

**Authors:** Marie-Francoise Noirot-Gros, Shalaka Shinde, Peter E. Larsen, Sarah Zerbs, Peter J. Korajczyk, Kenneth M. Kemner, Philippe H. Noirot

**Affiliations:** Biosciences Division, Argonne National Laboratory, Lemont, IL, United States

**Keywords:** biofilms, plant-root colonization, *Pseudomonas fluorescens*, *Laccaria bicolor*, *Populus tremuloides*, mycorrhization, mucilage

## Abstract

Rhizosphere-associated *Pseudomonas fluorescens* are known plant growth promoting (PGP) and mycorrhizal helper bacteria (MHB) of many plants and ectomycorrhizal fungi. We investigated the spatial and temporal dynamics of colonization of mycorrhizal and non-mycorrhizal Aspen seedlings roots by the *P. fluorescens* strains SBW25, WH6, Pf0-1, and the *P. protegens* strain Pf-5. Seedlings were grown in laboratory vertical plates systems, inoculated with a fluorescently labeled *Pseudomonas* strain, and root colonization was monitored over a period of 5 weeks. We observed unexpected diversity of bacterial assemblies on seedling roots that changed over time and were strongly affected by root mycorrhization. *P. fluorescens* SBW25 and WH6 stains developed highly structured biofilms with internal void spaces forming channels. On mycorrhizal roots bacteria appeared encased in a mucilaginous substance in which they aligned side by side in parallel arrangements. The different phenotypic classes of bacterial assemblies observed for the four *Pseudomonas* strains were summarized in a single model describing transitions between phenotypic classes. Our findings also reveal that bacterial assembly phenotypes are driven by interactions with mucilaginous materials present at roots.

## Introduction

Plant-growth-promoting (PGP) rhizobacteria and mycorrhizal fungi exert their beneficial effects on plants through direct and indirect interactions with roots, which lead to increased availability of soil nutrients to the plant, production of pathogen-antagonist metabolites, stimulation of plant systemic defenses, and increased plant resistance to biotic and abiotic stresses (Rodríguez and Fraga, 1999; Barea et al., 2005). In return, rhizosphere microorganisms acquire photosynthetically-derived carbon from the plant in form of sugars and organic acids. These interactions play a major role in shaping activities of rhizosphere communities that contribute directly to terrestrial plant biomass accumulation and carbon cycling and sequestration (Morgan et al., 2005; Compant et al., 2010; Cumming et al., 2015). An important part of these interactions is driven by microorganism colonization and persistence at roots (Varivarn et al., 2013; Dupuy and Silk, 2016). Although mycorrhizae is the most prevalent type of symbiosis with higher plants (Southworth, 2012), plants also engage in mutualistic associations with various advantageous microorganisms such as root-associated bacteria. More generally, in nature, plants form symbiotic communities comprising mycorrhizal fungi and beneficial bacteria (Frey-Klett et al., 2007; Churchland and Grayston, 2014; Mitter et al., 2016). Soil-borne *Pseudomonas* spp. have been identified as PGP bacteria, providing beneficial effects to multiple plant species (Rincon et al., 2005; Taghavi et al., 2010; Dominguez et al., 2012; Weston et al., 2012; Giles et al., 2014; Habibi et al., 2014; Pastor et al., 2014; Timm et al., 2015). *P. fluorescens* species are highly abundant in the root microbiome of *Populus* (Aspen) trees (Gottel et al., 2011; Brown et al., 2012; Weston et al., 2012; Timm et al., 2015), which are widely distributed in the Northern Hemisphere and establish symbiotic interactions with mycorrhizal fungi (Burns and Honkala, 1990; Lammers et al., 2004; Tuskan et al., 2004). The colonization of *Populus* rooted cuttings by a GFP-labeled endophytic *P. putida* revealed its presence in both root endosphere and rhizosphere (Germaine et al., 2004). In addition to *Populus* rhizoplane, many *P. fluorescens* form biofilms on the hyphal surface of the ectomycorrhizal fungus *Laccaria bicolor* (Frey-Klett et al., 2007; Timm et al., 2015, 2016). Numerous rhizobacteria, including *P. fluorescens* have been shown to improve mycorrhizal formation, and consequently, are considered mycorrhizal helper bacteria (MHB) (Garbaye, 1994; Founoune et al., 2002; Frey-Klett et al., 2007; Labbé et al., 2014).

Previous works indicate that Pseudomonads colonize roots under a wide range of conditions predominantly by forming biofilms (Spiers and Rainey, 2005; Huang et al., 2007; Koza et al., 2009; Barahona et al., 2010; Mann and Wozniak, 2012; Martin et al., 2016). Biofilms are surface-adhered assemblies of microorganisms embedded within a multicomponent exopolysaccharide (EPS)-containing matrix (Davey and O'Toole et al., 2000). This surrounding matrix shapes the whole bacterial edifice, plays an important role in adaptation to various ecological niches, and provides protection from environmental threats (Spiers and Rainey, 2005; Koza et al., 2009; Mann and Wozniak, 2012). On the rhizoplane, biofilms form protective micro-environments allowing the localized diffusion of nutrients and metabolic compounds (Bogino P. C. et al., 2013; Bogino P. et al., 2013). Biofilm formation at plant roots is triggered by physico-chemical cues, such as water and nutrient availability, and involves bacteria-bacteria communication (Martins et al., 2014; Zúñiga et al., 2017). In many Gram-negative bacteria, quorum sensing (QS) plays a pivotal role in biofilm formation through the production and sensing of small diffusible autoinducer (AI) molecules, such as N-Acyl homoserine lactones (AHLs) that monitor cell density and regulate cell behaviors (Newton and Fray, 2004; Waters and Bassler, 2005). Plants also evolved to sense bacterial AIs and respond by secreting plant metabolites that act on bacterial QS activities (Gao et al., 2003; Schikora et al., 2016). Under laboratory conditions, *Pseudomonas* was found to accumulate around the cap of wheat roots grown in transparent soil, as well as within the nutrient-rich interstitial spaces at intercellular junctions on the rhizoplane (Downie et al., 2014). Other studies observed *Pseudomonas* colonies on upper parts of the roots (ChinAWoeng et al., 1997; Bloemberg et al., 2000; Unge and Jansson, 2001; Humphris et al., 2005). Most root colonization studies use model plants such as Arabidopsis and tomato observed during the early phases of seedling development (Gamalero et al., 2004). Generally, the plant seedlings are grown in gnotobiotic systems and root colonization is observed at a specific time point after bacterial inoculation, revealing the status of colonization at this time but missing the temporal dynamics. Investigation of the spatial and temporal patterns of colonization over an extended time after inoculation is needed to help understand the complex relationships between plant-microbe interactions and PGP.

Recently, strain-specific PGP activities of three *P. fluorescens* strains SBW25, WH6, Pf0-1, and *P. protegens* strain Pf-5 were described using *Populus tremuloides* Michx. (trembling Aspen) seedlings grown in vertical agar plates (VAP) for 5 weeks (Shinde et al., 2017). However, the colonization patterns exhibited at Aspen root seedling by the different strains was not assessed. SBW25, initially isolated from the phyllosphere of a sugar beet plant (Rainey and Bailey, 1996), efficiently colonizes the rhizosphere of many plants (Unge and Jansson, 2001; Gal et al., 2003; Humphris et al., 2005; Jackson et al., 2005; Wilton et al., 2017). WH6 originates from the rhizosphere of wheat and produces a large array of secondary metabolites with biocontrol activities (Kimbrel et al., 2010). WH6 and SBW25 are phylogenetically close and have a similar genomic organization (Kimbrel et al., 2010). *P. fluorescens* Pf0-1, isolated form a loam soil (Compeau et al., 1988), is a model for the study of bacterial behavior and fitness in soils (Silby et al., 2009; Varivarn et al., 2013). *P. protegens* strain Pf-5, originally classified as a *P. fluorescens*, has been isolated from the cotton rhizosphere (Howell and Stipanovic, 1979) and possesses plant beneficial traits (Paulsen et al., 2005; Bruto et al., 2014). Genomic comparison of SBW25, Pf0-1, and Pf-5 revealed considerable divergence. A high percentage of the plant-inducible genes (42%) are not shared between the strains, suggesting specialized functions (Silby et al., 2009). These strains also possess many genetic determinants for biofilm formation including the pathways controlled by nucleotide second messenger signaling (Gal et al., 2003; Paulsen et al., 2005; Spiers and Rainey, 2005; Koza et al., 2009; Silby et al., 2009; Kimbrel et al., 2010; Newell et al., 2011; Boyd et al., 2012; Varivarn et al., 2013; Martins et al., 2014; Ayub et al., 2015; Hengge et al., 2016). Knowledge about quorum sensing (QS) and auto-inducer pheromone molecule remains sparse for these strains as SBW25 does not produce an AHL-type of auto-inducer (Unge and Jansson, 2001; de Bruijn and Raaijmakers, 2009; Martins et al., 2014). However, these strains possess homologs of well-known QS systems such as the LuxR-family of gene regulators (de Bruijn et al., 2008; Mastropaolo et al., 2012).

Here, we characterize the temporal dynamic and colonization patterns of Aspen roots by four *P. fluorescens* strains with previously described PGP activities (Shinde et al., 2017). We seek to investigate potential relationships between colonization profiles and beneficial traits for the plant. To this end, we engineered SBW25, WH6, Pf0-1, and Pf-5 to express a codon-optimized mNeonGreen fluorescent protein (Wilton et al., 2017). Colonization of Aspen roots by fluorescently-labeled *P. fluorescens* strains was imaged over the course of 5 weeks, using the VAP system under replete nutrient condition for the plant (Shinde et al., 2017). In this system, the only source of carbon available to the bacteria are sugars and organic acids produced by plant photosynthesis, allowing us to monitor bacterial colonization and survival strategies that effectively depend on interactions with the plant. Experiments were conducted on plant-roots alone or inoculated with the ectomycorrhizal fungus *L. bicolor*. We found that *Pseudomonas* strains adopted a range of morphological phenotypes on the rhizoplane, ranging from micro-colonies to highly structured biofilms, with temporal patterns depending on the bacterial strain and community composition. In our system, *P. fluorescens* SBW25 and WH6 formed dense biofilms and PGP phenotypes, suggesting that biofilm formation and PGP activities can be associated. Knowledge of the behavior and organization of bacteria on plant root surfaces could help develop multi-organism associations that improve sustainable plant feedstock production.

## Materials and methods

### Aspen tree seedlings, fungus, and bacterial resources

*Populus tremuloides* Michx. (trembling Aspen) seeds were obtained from the National Tree Seed Center, Natural Resources Canada, Fredericton NB, Canada. Strain *P. protegens* Pf-5 was obtained from ATCC (American Type Cell Culture) Catalog# BAA-477], and *P. fluorescens* strains Pf0-1(Garbeva et al., 2011), SBW25 (Preston et al., 2001), and WH6 (Banowetz et al., 2008) were provided by the cited laboratories (Supplementary Table S1). *Laccaria bicolor* (strain S238N, obtained from ATCC) was cultured and maintained on Modified Melin Norkan's (MMN) media at 20°C, as described in Larsen et al. (2016).

### *Pseudomonas* strains labeling

*Pseudomonas fluorescens* strains SBW25, WH6, and Pf0-1 were genetically labeled by inserting a DNA cassette coding for the mNeongreen (mNG) fluorescent protein (Shaner et al., 2013), expressed from a constitutive promoter (Pc) combined with tetracycline resistance genes, into the chromosome of each strain (see Supplementary Figure S1, Supplementary Tables S2-S4). *P. protegens* Pf-5 was labeled by insertion of the mTurquoise (mTO) fluorescent protein expressed from the constitutive Pc promoter and associated with a gentamycin resistance gene (Supplementary Figure S1, Supplementary Tables S2-S4). Briefly, insertion sites were selected within non-coding intergenic regions and designed to minimally interfere with expression of flanking coding sequences. The 1kb-long chromosomal regions flanking the targeted insertion site were PCR-amplified and joined to the cassette by assembly cloning methods. The resulting DNA fragments were introduced by electroporation in the cognate *Pseudomonas* strains carrying a plasmid-borne RecET-like recombineering system (Swingle et al., 2010; see Supplementary Figure S1). Homologous integration of the cassette was selected by plating SBW25, WH6, and Pf01 cells on LB plates supplemented with tetracycline, and Pf-5 cells on LB supplemented with gentamycin. The chromosome structure of each construct was verified by PCR using combinations of distal and internal primer pairs (Supplementary Figure S1). Verified strains were propagated on LB medium without selection to isolate plasmid-free cells.

### Time-course study of Aspen root colonization by pseudomonas in vertical agar plates

Seeds were surface sterilized by treating with a 2% Tween/2.5% Na hypochlorite solution followed by several washes in sterile water, as previously described (Shinde et al., 2017). Sterilized seeds were left in water in the dark overnight prior to transfer into a jar containing 1% of phytablend for germination. Germinated seedlings were then transferred in vertical plates, containing or not the ectomycorrhizal fungus *L. bicolor*, as previously described (Shinde et al., 2017). For each condition, individual seedlings were inoculated with a fluorescently-labeled *Pseudomonas* strain. Three individual seedlings were sampled for microscopic and macroscopic examinations each week, for up to 5 weeks. The experimental procedure is illustrated in Supplementary Figure S2.

#### Vertical agar plate assay

Vertical agar plate (VAP) assay was performed as previously described (Shinde et al., 2017). Bleach-sterilized Aspen seedlings were first germinated 7 days in agar-containing jars and transferred in vertical plates on cellophane-coated agar media (4 mM NH_4_NO_3_,1 mM CaSO_4_, 1.5 mM K_2_SO_4_, 0.5 mM MgSO_4_, and 1.5 mM KH_2_PO_4_, adjusted to pH = 5.6, 1% agar). For Aspen mycorrhizae, *L. bicolor* was first grown on P20 media as previously described (Müller et al., 2013), and agar plugs containing the fungus were placed on the cellophane surface and incubated in a dedicated growth chamber at 30°C for a week, at which point 5–6 Aspen seedlings were transferred on each plate at ~2 cm intervals (Supplementary Figure S2). Plates were incubated at a 75° angle for a week and Aspen roots were inoculated with *Pseudomonas* strains. Plates were maintained in same growth conditions up to 5 weeks after bacterial inoculation.

#### Bacteria-fungus-root communities

Fluorescently-labeled *Pseudomonas* were grown overnight in LB supplemented with the appropriate antibiotic. For each culture, OD_600_ was measured, cells were washed and re-suspended in phosphate-buffered saline, pH 7.4. Prepared cell suspension used for inoculum was adjusted to an OD_600_ of 4, and 10 μl aliquots were deposited on the primary root of each Aspen seedling (non-mycorrhizal and mycorrhizal). This inoculum contained about 4 × 10^9^ colony forming units, as measured by serial dilution and plating experiments. Each week, three seedlings per inoculated *Pseudomonas* strain were sampled for microscopic observations of the roots and for bacterial numeration and biomass determination as described in Supplementary Figure S2.

#### Biomass and bacterial numeration

For each seedling, the whole root and shoot systems were separated and were weighted separately (fresh weight). Each root system was then placed individually in a 2 ml microcentrifuge tube containing 1.5 ml of PBS and subjected to sonication at 40 kHz for 5 min in a water filled sonication bath. This sonication setting was described to separate microorganisms from Aspen rhizoplane (Utturkar et al., 2016). The sonicated roots were then examined by fluorescence microscopy to assess the proper dissociation of bacteria. Sonication was found to efficiently detach bacterial cells in all conditions for all strains, except for mycorrhizae colonized by SBW25 at week 1, where some cell masses were found to remain resistant to even extended sonication time. The bacteria dissociated from roots were then enumerated by serial dilution and plating on LB plates containing the appropriate antibiotic.

#### Microscopic observations and imaging

Each week, three seedlings were taken for the microscopic observation of root colonization by bacteria. Whole root systems were mounted on slides in PBS buffer containing 15% glycerol, and observed using a spinning disc confocal microscope using a Nikon Eclipse Ti-E coupled with CREST X-Light^TM^ confocal imager, equipped with objectives Nikon CFI Plan Fluor 10X, DIC, 10x/0.3 NA (WD = 16 mm), a Plan Apo 60x /1.20 NA WI water (WD = 0.22 mm) and a Plan Apo λ 100x/1.45 NA oil (WD = 0.13 mm). Mycorrhizal roots were first covered with 0.05% calcofluor white solution (in PBS) for 10 min prior to transfer on the slide. To reconstruct the root surface topology, Z-stack images were recorded with excitations 470 nm (mNG) and 555 nm (plant red autofluorescence), as well as 395 nm (calcofluor) when necessary. Images were collected over 10–20 μm depth with 0.2 μm (x100) or 0.3 μm (x60) steps. mTO was recorded at 440 nm (cyan) or 470 nm (green). After collection images were false-colored to colors corresponding with recorded emission wavelength via the NIS-Elements Imaging Software.

### Statistical analysis

Aspen seedling phenotype data were analyzed using students *t-*test with a statistical significance of *P* < 0.1. Data are summarized in Table 1.

**Table 1:** Plant root and shoot mass measurements after 4 weeks.

		**Sht**	**SD**	***P***	**Rt**	**SD**	***P***
Non-mycorrhizal	Control (3)	9.4	5.2		2.8	1.2	
	SBW25 (2)	21.3	2.5	0.064^+^	9.9	2	0.076^+^
	Pf0-1 (2)	4.5	0.5	0.12	4.7	1.3	0.119
	WH6 (3)	16.6	4.4	0.073^+^	3.8	2.1	0.26
	PF5 (3)	8.3	2.4	0.389	3.4	0.9	0.281
Mycorrhizal	Control (3)	25.5	11.8		5.7	4.1	
	SBW25 (3)	23.5	7.9	0.409	12.4	8.6	0.15
	Pf0-1 (2)	19.7	2.3	0.246	3.9	1.7	0.273
	WH6 (3)	27.5	6.2	0.404	8.8	4.7	0.222
	PF5 (3)	23.9	13.4	0.443	13.6	6.1	0.07^+^

Control is no-bacteria inoculated condition. Shoot (Sht) and Root (Rt) weights are in mg. The number of measured samples for biomass is indicated in parenthesis. Standard deviation (SD) and P-values (P, t-test) are indicated. (

+ *) not significant but relevant differences 0.05 < p < 0.1*.

### Phenotype transition modeling

To capture the diversity and progression of colonization patterns at the roots, we generated a phenotype transition model. The dynamic changes in the observed bacterial microscopy phenotypes could be visualized as a network in which transition between phenotypes were drawn as a “map” of the most probable paths from initial to terminal states of bacterial structures observed at the roots and of the connection between them. This model utilizes the phenotypic observations of bacterial structures from Table 2, aggregated across all strains and mycorrhizal conditions. The model assumes that all strains are potentially capable of forming all observed biofilm types. In the model, nodes are the 10 observed colonization phenotypes. The size of the node is proportional to the frequency at which that phenotype has been observed. Edges in this network are the transitions in observed bacterial structures from one time point to the next. In cases where more than one phenotype was assigned to a single observation, transition to or from observed instances was counted as one-half an observation. Edges are weighted according to the frequency of observations, normalized such that the weights of the out-degree edges of a node sum to 100%. The network was drawn using Cytoscape 2.8.1 (http://cytoscape.org/).

**Table 2:** Summary of bacterial patterns observed at non-mycorrhizal **(A)** and mycorryhizal **(B)** Aspen roots over 5 weeks.

	**SBW25**	**Pf0-1**	**WH6**	**Pf-5**
**(A) NON-MYCORRHIZAL**				
W1	LS	NP/F	C	NP
W2	LP	NP	LP/DBS	NP
W3	LP/SP	SP	LS	LS/IC
W4	LP/SP	SP	SP	LC
W5	DBS	–	SP	–
**(B) MYCORRHIZAL**				
W1	DBS	NP/F	C	NP
W2	SP	NP	LP/DBS	DS
W3	SP	–	–	–
W4	–	–	–	–

*No pattern (NP), long strips (LS), long patch macrocolonies (LP), short patch microcolonies (SP), high bacterial density coating (C), dense biofilm structures (hives and/or bulges like structures) (DBS), filamentous cells (F), intracellular (IC), around loose cap cells (LC), and no (or too few) bacteria (–)*.

## Results

### Temporal dynamics of colonization of non-mycorrhizal and mycorrhizal Aspen roots by different *Pseudomonas* strains

**Figure 1:**
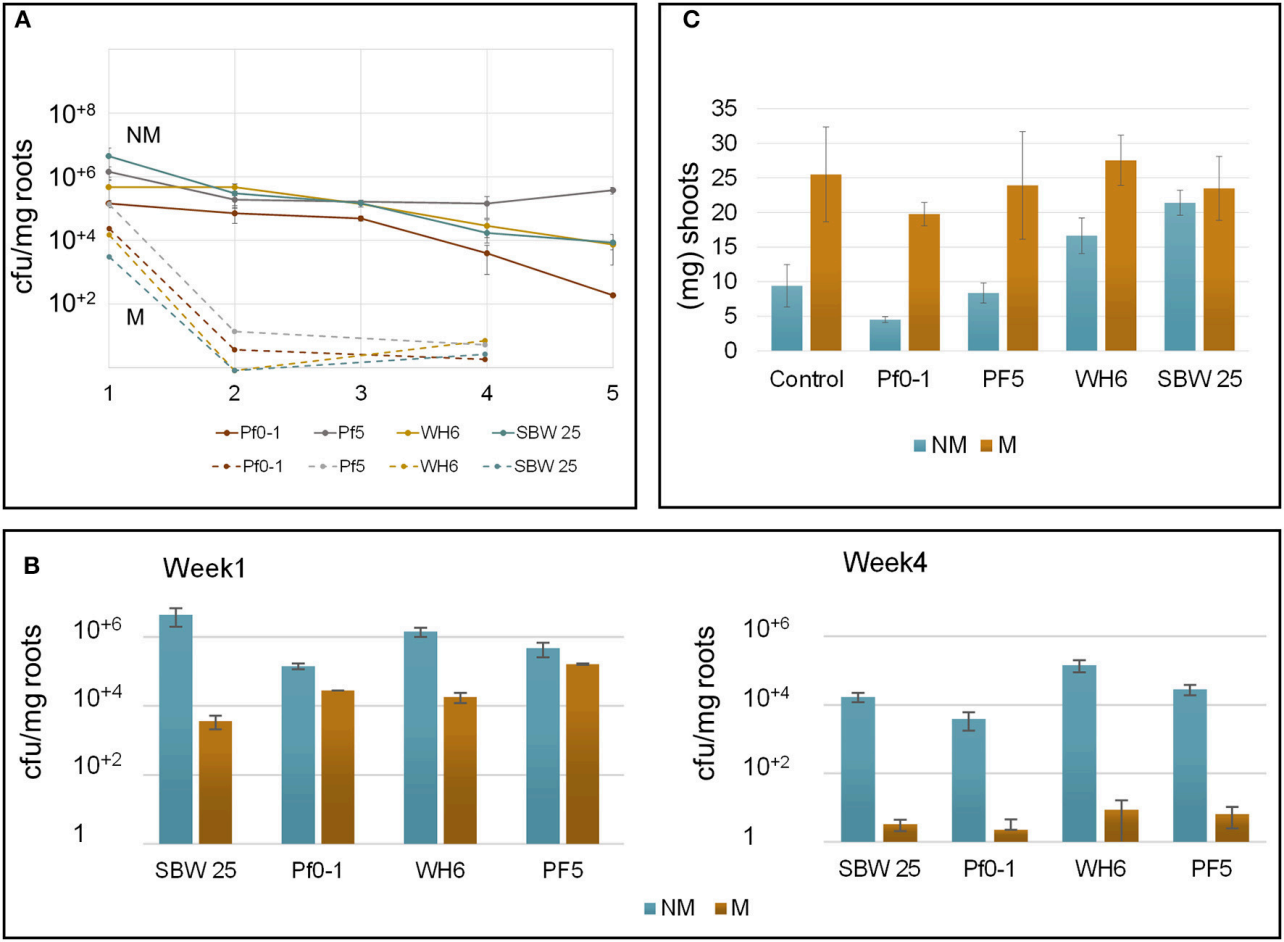
Parameters describing *Aspen-Pseudomonas* interactions in the vertical plate assay. **(A)** Monitoring the persistence of bacteria at Aspen roots over 5 weeks (x-axis). Cell counts were normalized per mg of roots (y-axis). Different *P. fluorescens* strains are specified by colors Cell count at non-mycorrhizal roots are indicated by plain circle and lines. Closed circle represent results from non-mycorrhizal plants, open circles represent results from plants colonized by *L. bicolor*. Vertical bars are standard errors of the means **(B)** Bacteria counts per mg of root biomass on non-mycorrhizal (NM) and mycorrhizal (M) roots after 4 weeks. **(C)** Plant growth promotion effects evaluated by monitoring the shoot biomass at week 4.

Bacterial colonization and viability at Aspen seedlings roots were explored over a period of 5 weeks after bacterial inoculation (Figure 1A). From the initial inoculum (~4 × 10^9^ bacteria), the number of viable bacterial cells recovered from non-mycorrhizal roots after 1 week was 5 × 10^6^ bacteria per mg of root for SBW25 and Pf-5 and 5 × 10^5^ for WH6 and Pf0-1. Colony counts remained stable for 2-3 weeks near ~10^5^ bacteria per mg of root for all strains. At 5 weeks, viable cells declined to ~10^4^ bacteria per mg of root for SBW25 and WH6 and 10^2^ bacteria per mg of root for Pf0-1, while Pf-5 maintained colony counts at 10^5^ (Figure 1A). Here, we monitored the cells present on the rhizoplane that dissociated upon sonication but did not take into account the bacterial cells in vicinity of roots that are not physically associated with the rhizoplane (i.e., the ecto-rhizosphere) and the bacteria within plant tissues (i.e., the endo-rhizosphere). Additionally, the sonication procedure detached most bacteria from the root surface and only occasional bacterial cells could be observed by microscopy on non-mycorrhizal roots after sonication. On the whole, all our strains persisted at the surface of Aspen roots during 5 weeks, maintaining a rather stable viable count during a period of 4 weeks.

Bacterial colonization of mycorrhizal roots differed from patterns observed with non-mycorrhizal roots (Figure 1B). Prior to bacterial inoculation of Aspen seedlings, the extent of root mycorrhization was assessed by microscopy, revealing that most roots were completely covered by a fungal veil (Supplementary Figure S3, see below). A week after inoculation by *P. fluorescens*, the number of bacteria recovered from mycorrhizal roots by sonication was reduced about 10- to 30-fold for Pf0-1, WH6, and Pf-5 and nearly 1,000-fold for SBW25. A few biofilms-like masses of SBW25 cells were observed to remain associated with the roots after sonication. These biofilms resisted to extended sonication times indicating that the bacterial cells were part of structures firmly associated to mycorrhizal roots. Such structures were never found at mycorrhizal roots after week 2. These observations imply that the number of SBW25 cells associated with mycorrhizal roots after 1 week is underestimated. After 4 weeks, the number of root-associated bacteria declined further to the range of 2-10 viable cells per mg of root in all strains (Figure 1B). This observation suggests that under our conditions, extensive ectomycorrhizal colonization by *L. bicolor* strongly interferes with colonization by *Pseudomonas* and hinders the capacity of bacterial cells to persist on the rhizoplane.

We assessed the effects of *Pseudomonas* and *Laccaria* on plant growth under our experimental conditions, by examining the shoot and root mass of Aspen seedlings (Figure 1C, Table 1). At week 4, mycorrhization of Aspen seedlings with *Laccaria* increased shoot (2.0-fold) and root biomass (2.0-fold) significantly [*p*-values 0.003 and 0.0186, *t*-test between all NM (*n* = 13) and M samples (*n* = 14), respectively]. This observation provides a good indication that the ecto-mycorrhization of Aspen roots in our VAP system is functional. We observed that co-culturing Aspen seedlings with *Pseudomonas* and *Laccaria* did not have a significant effect on Aspen biomass, relative to *Laccaria* alone. In non-mycorrhizal condition, *Pseudomonas* SBW25 and WH6 exhibited a positive effect on Aspen seedling shoot and root biomass while the effects of Pf0-1 and Pf-5 inoculation were similar to the control (Table 1). Despite of the small size of samples used in this study, our observations remained consistent with previous work by Shinde et al. (2017) in which PGP activities were higher for SBW25 and WH6 relative to Pf0-1 and Pf-5 in the same assay (Shinde et al., 2017).

### Colonization patterns of *Pseudomonas* at non-mycorrhizal Aspen roots

**Figure 2:**
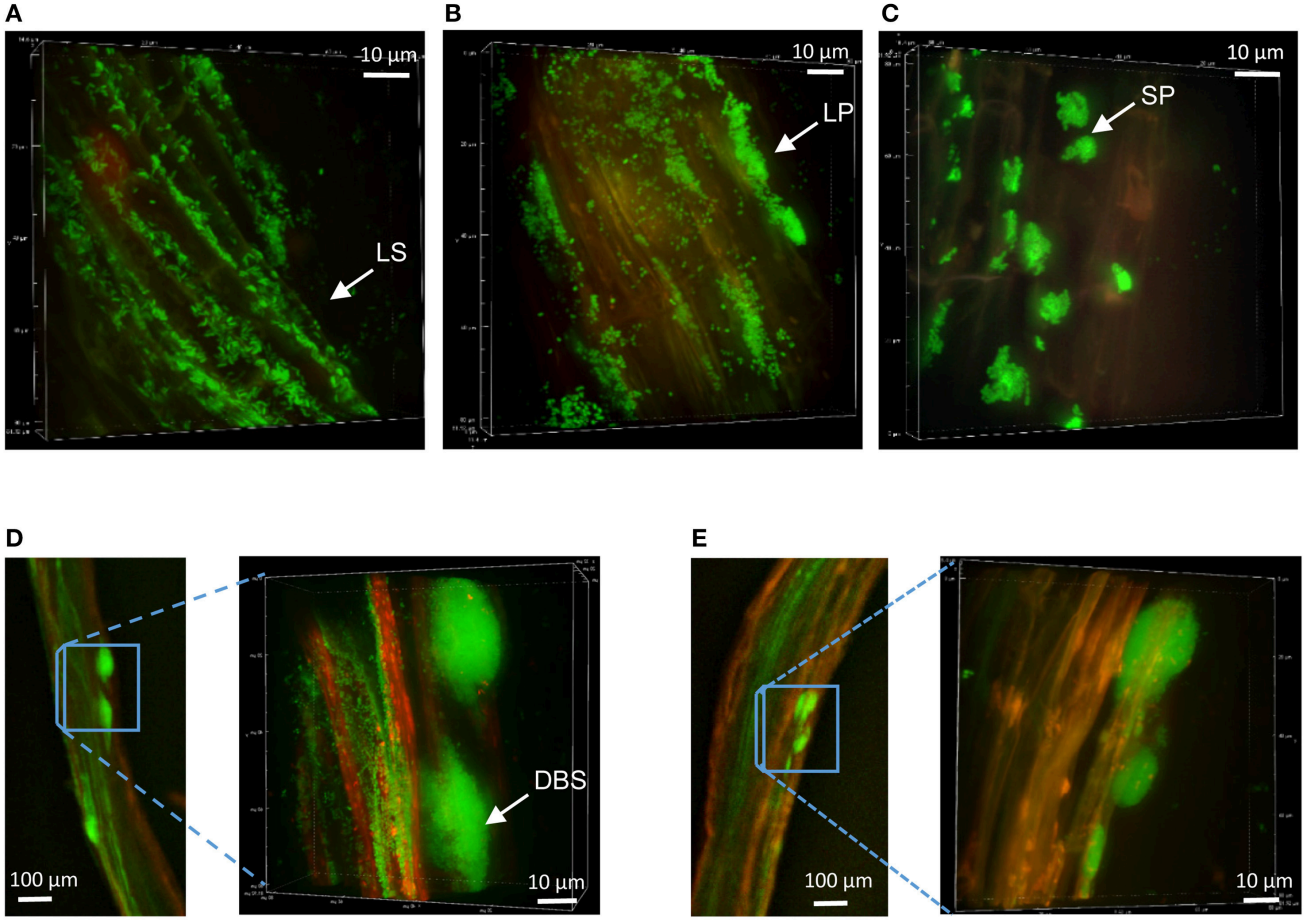
Colonization patterns of *P. fluorescens* strain SBW25 on non-mycorrhizal Aspen roots. Z-stack reconstruction of root surface topology was performed by SDCM at x100 magnification. SBW25 cells are green and plant tissues are visualized using red auto-fluorescence. Scale is indicated by a white bar. Images are representative of colonization patterns on all the observed plant roots. **(A)** Long strip (LS) colonization pattern observed 1 week after inoculation, **(B)** long patch (LP) patterns after 2 weeks, **(C)** short patch (SP) microcolonies formed after 3 weeks, **(D,E)** bulge–like structures observed along roots after 4-5 weeks, with enlarged images showing dense biofilm-like structures (DBS) in which cells appear encased in a matrix.

**Figure 3:**
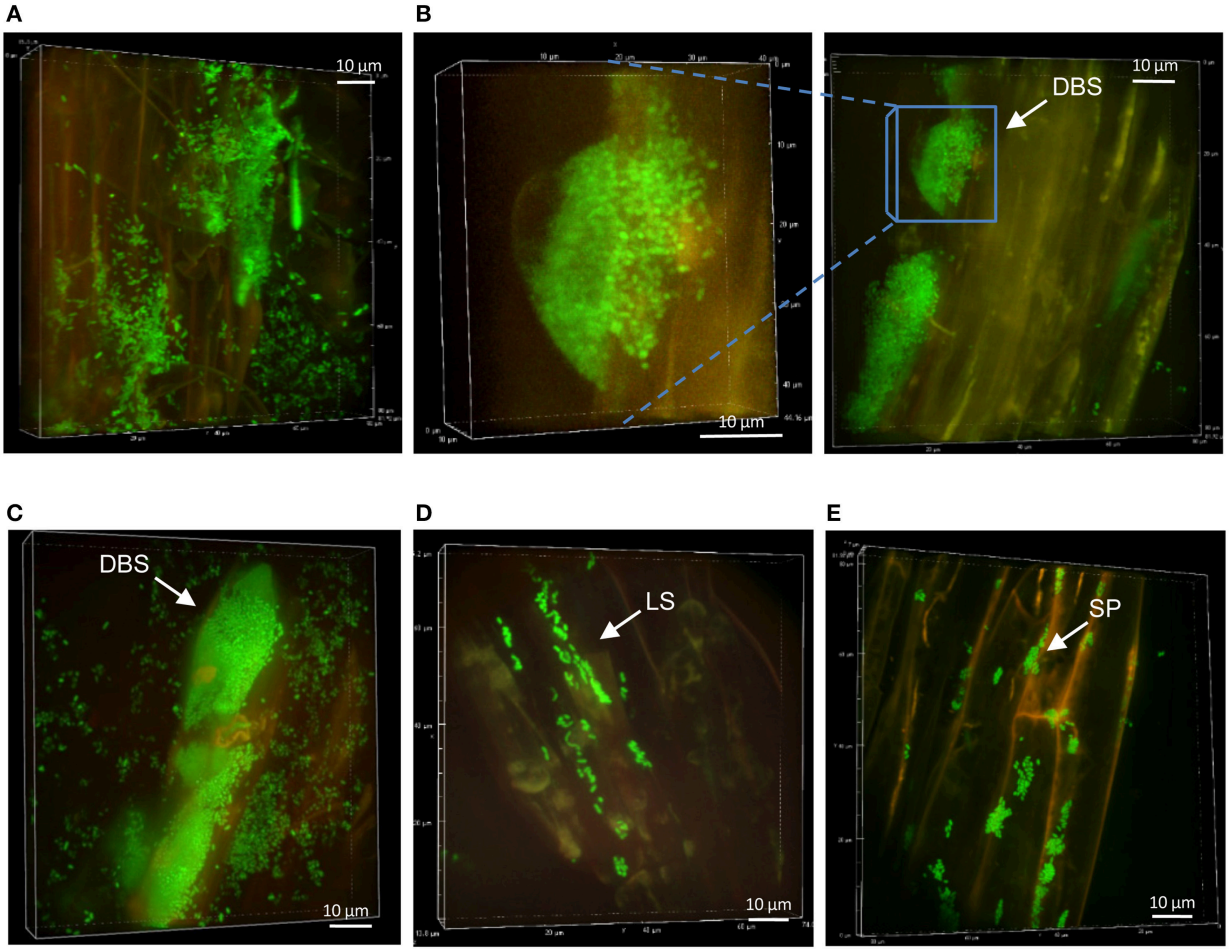
Colonization patterns of *P. fluorescens* strain WH6 on non-mycorrhizal Aspen roots. Z-stack reconstruction of root surface topology were performed by SDCM at x100 magnification. WH6 cells are green and plant tissues are visualized using red auto-fluorescence. Images are representative of colonization patterns on all the observed plant roots. Scale is indicated by a white bar. **(A)** Bacterial colonization at week 1, **(B,C)**, morphology of hive-like structures formed at week 2, **(D)** LSs observed at week 4, and **(E)** SP microcolonies persisting at week 5.

**Figure 4:**
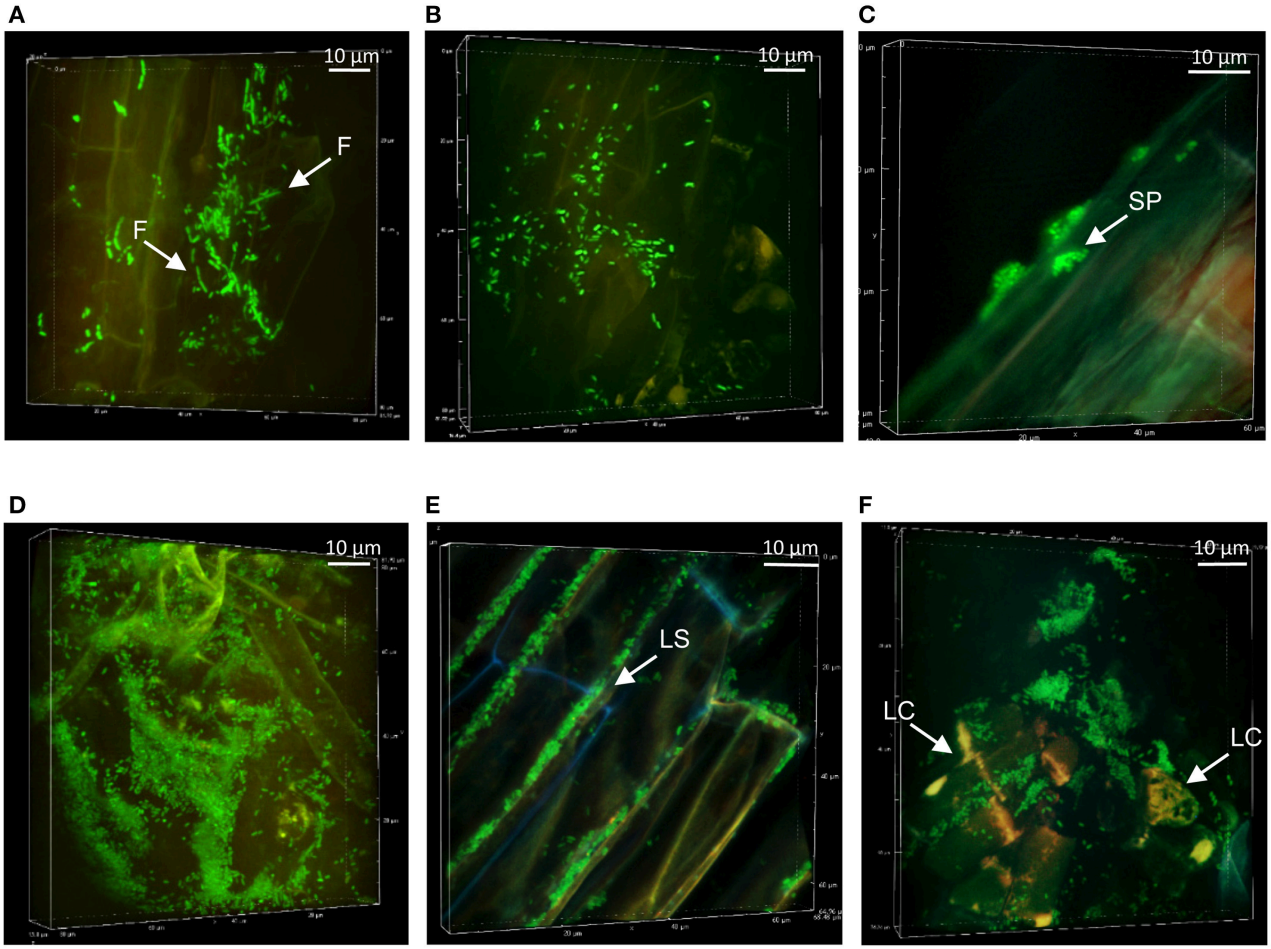
Colonization patterns of *Pseudomonas* strains Pf0-1 and Pf-5 at non-mycorrhizal Aspen roots: Z-stack reconstruction of root surface topology were performed by SDCM at x100 magnification. Bacterial cells are green and plant tissues are visualized using red auto-fluorescence. Images are representative of colonization patterns on all the observed plant roots. Pf0-1 morphology and structures after week 1 **(A)**, week 2 **(B)**, and week 4 **(C)**. Elongated filamentous cells are indicated by white arrow heads. Pf-5 colonization patterns at week 1 **(D)**, at week 3 with colonization of the interstitial space between plant cells **(E)**, and at week 4 **(F)**. Note that here that the mTO-labeled Pf-5 have been observed with similar excitation/ emission settings as used for the mNG.

The colonization patterns of Aspen roots by *Pseudomonas* strains SBW25, Pf0-1, Pf-5, and WH6 expressing fluorescent protein (FP) reporters were examined by SDCM every week during a period of 5 weeks. For the FP-labeled SBW25 strain, a continuous rearrangement of bacterial cells on the surface of expanding roots was observed over time. Patterns corresponding to long strips (LS, Figure 2A, Supplementary Movie S1), and long patch (LP) macrocolonies (Figure 2B) were observed covering the first third of the root above the root tips, up to 2 weeks after inoculation and were followed by the appearance of short patch (SP) microcolonies at week 3 (Figure 2C). Dense biofilm-like structures (DBS), mostly localized in the second third of the root length (from the tips) were observed at week 5, (Figures 2D,E). These typical patterns were present at all observed plant roots. Similar colonization patterns were observed with other strains but with a different chronology. The FP-labeled WH6 strain was found to coat densely the rhizoplane at week 1 (Figure 3A), and to form dome-shaped DBS looking like bee hives at week 2 (Figures 3C,D). These DBS receded in LS at week 3 and then in SP after 4-5 weeks. In contrast, no dense biofilm-like structure was formed by the Pf0-1 and Pf-5 strains. Pf0-1 did not exhibit any specific colonization pattern during 3 weeks (Figures 4A,B), but formed SP microcolonies at week 4 (Figure 4C). Interestingly, we observed the presence of numerous elongated bacterial cells at week 1, suggesting exposure to stress with transient formation of elongated cells (Figure 4A). Finally, Pf-5 colonization included a dense coverage of all the rhizoplane by bacteria at week 1-2 (Figure 4D), followed by a preferential occupancy of interstitial spaces between epidermal root cells (Figure 4E). In addition, Pf-5 bacterial assemblies were observed at root tips around the loose cap cells until week 3 (Figure 4F). These results are summarized in Table 2A.

### Bacterial colonization pattern at mycorrhizal Aspen roots

*L. bicolor* is known to colonize Aspen by sheathing the roots within a dense mycelia mat (Felten et al., 2009). We examined this interaction between *Laccaria* and Aspen roots in our VAP assay, which leads to functional ectomycorrhizae promoting plant growth (Figure 1C). Mycorrhizal roots were dyed with calcofluor white prior to SDCM observation. A mantle-like structure formed by the fungus covered the whole length of the root up to the root tips (Supplementary Figure S3). Time course examination of the colonization patterns by the different *Pseudomonas* strains was possible up to 2 weeks after inoculation, in agreement with cell titrations (Figure 1B). However, the rapid decrease of bacterial populations at mycorrhizal Aspen roots hindered our ability to collect multiple independent observations of bacteria-containing structures.

**Figure 5:**
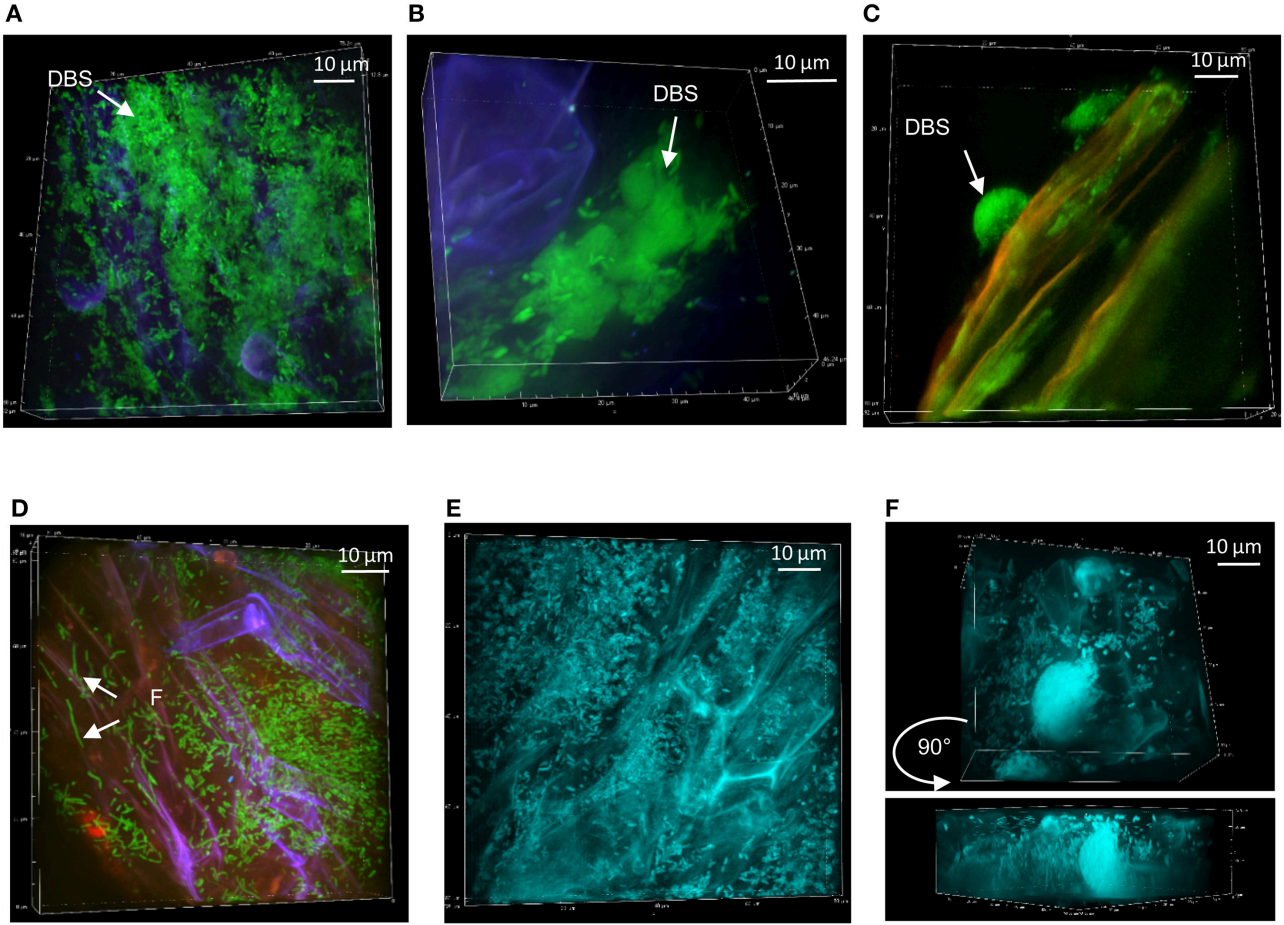
Persistence of *Pseudomonas* strains at mycorrhizal Aspen roots: **(A)** SBW25 (green) on mycorrhizal Aspen root (red plant and blue fungus) at week 1. **(B)** Dense SBW25 forming structures encased within the fungi mantle are revealed after sonication. **(C)** WH6 forming highly dense hemispherical structure at week 2. **(D)** Pf0-1 bacterial cells on calcofluor-stained mycorrhized roots at week 1. **(E)** Dense colonization by mTO-labeled Pf-5 at week 1, and **(F)** at week 2, showing the presence of DBS. Side view revealing vertical positioning of bacteria.

**Figure 6:**
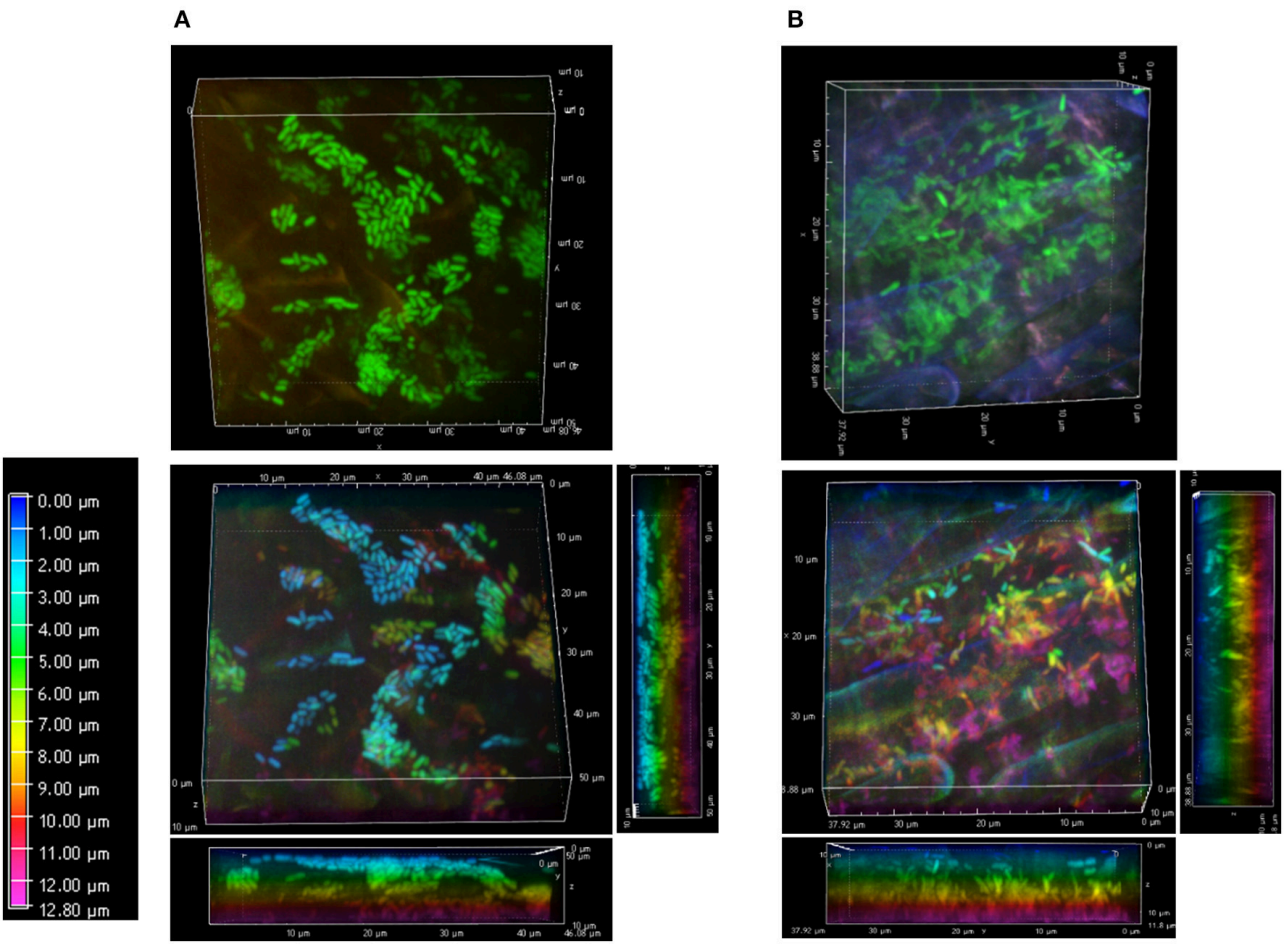
Bacterial arrangements at root surfaces. Surface arrangements of SBW25 cells at non-mycorrhizal **(A)** and mycorrhizal **(B)** roots. 3D reconstruction of fluorescence (top panels) and the same images in which cells are false-colored according to their depth in the reconstructed volume (bottom panels). Depth color scale is the left-most panel. **(A)** Side view projections of the whole volume show that the pattern and alignment of SBW25 cells does not change substantially with depth relative to the surface of non-mycorrhizal roots. Most bacteria are close to the top surface of the 3D-volume (false-colored in blue and green). **(B)** On mycorrhizal roots, SBW25 cells appear encased in a layer of colloidal substance in which most cells are not anymore on the top but deeper inside the volume (false colored yellow and pink) and oriented perpendicularly to the root surface.

At week 1, FP-labeled SBW25 appeared dispersed over the fungal veil, sometimes embedded within it, and formed some DBS (Figure 5A). Interestingly, these DBS assemblies remained associated with the mycorrhizal sheath after sonication (Figure 5B), suggesting that bacterial cells may be trapped in a cohesive matrix in presence of the fungus. However, these cohesive structures did not account for the sharp decrease in the count of bacteria released from fungal roots because no fluorescent bacteria could be detected on sonicated fungal roots beyond week 2. Side-view projection of reconstructed 3D volumes showed that at week 1, SBW25 cells are embedded in a fungus-associated mucilaginous layer. Most bacterial cells appear to lie deep in the volume and to adopt an orientation perpendicular to the root surface as depth increases (Figure 6B). In absence of the fungus, bacterial cells are predominantly in the top part of the volume and exhibit no specific orientation relative to the root surface (Figure 6A).

At week 2, strain WH6 formed LPs and DBSs at mycorrhizal roots, as it did on non-mycorrhizal roots (see Figure 3). Striking structures of perfect hemispheric shape (Figure 5C) were observed, suggesting a complex interplay between biomechanical forces and the surrounding matrix. With strain Pf0-1, cells densely covering mycorrhizal roots with no specific pattern were observed at week 1, which included some filamentous cells (Figure 5D). Similar patterns were observed for strain Pf-5 (Figure 5E), with a few DBS forming at week 2 (Figure 5F). We did not observe Pf-5 forming DBS on non-mycorrhizal roots, suggesting that DBS formation is induced by the presence of *Laccaria*. These results are summarized in Table 2B. Overall, *Pseudomonas* strains SBW25, WH6, and Pf-5 but not Pf0-1 formed dense biofilm-like assemblies at mycorrhizal Aspen roots. However, these DBS trapped in the fungus did not persist after 2 weeks. Of note, SBW25 produced DBS structures earlier (week 1) on mycorrhizal roots than on non-mycorrhizal roots.

### Internal architecture of *Pseudomonas* biofilms

The diversity of structures formed during colonization of Aspen roots by different *Pseudomonas* strains and the observation that bacterial cells appear to be encased in a matrix-like substance in the presence of the fungus prompted us to examine in more details the structural and spatial organization of bacterial cells within the different type of assemblies. We focused our attention on the different biofilm-like structures on the root surface and on the colloidal-like material that contained bacteria at the surface of mycorrhizal roots, and on the plant mucilage produced at root tips.

**Figure 7:**
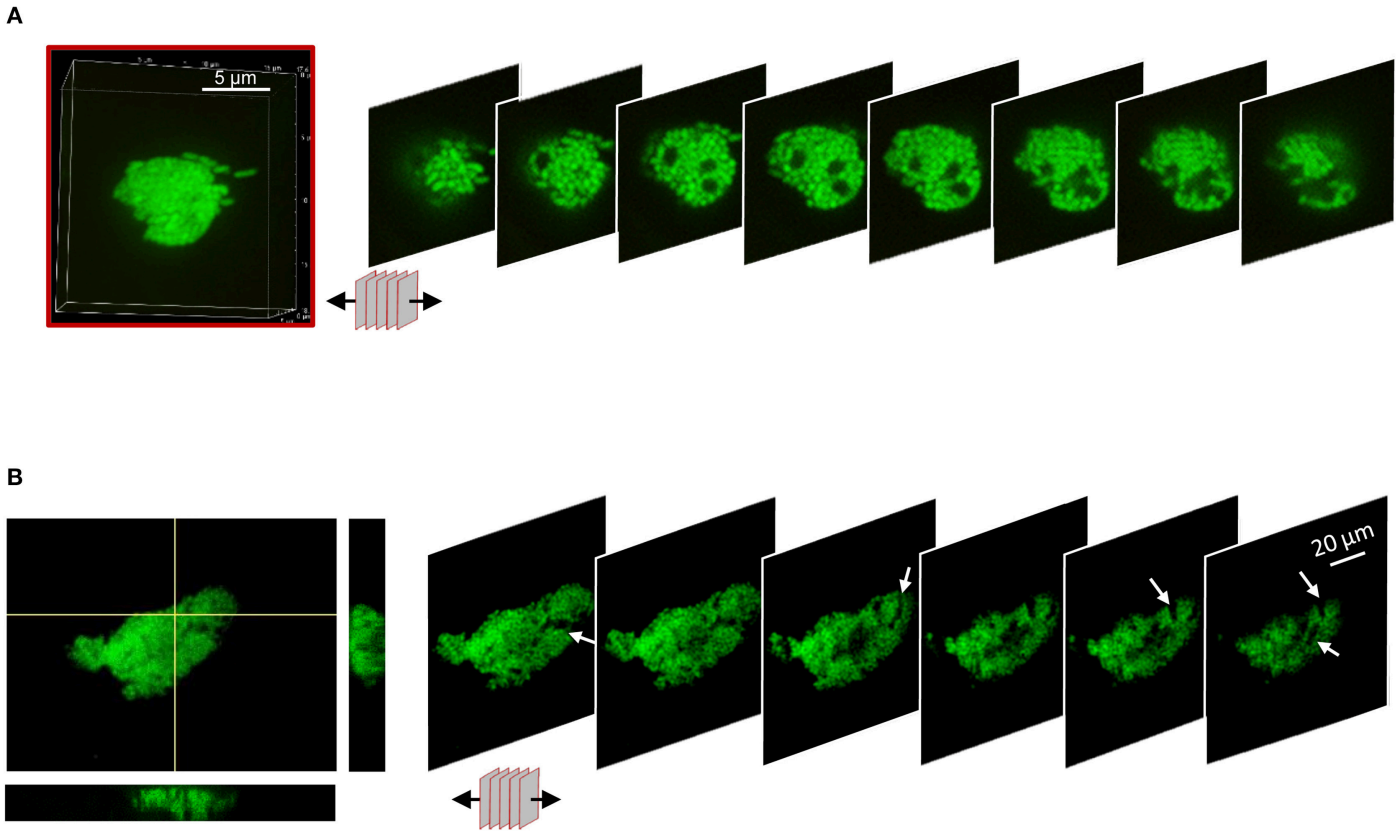
Internal architecture of bacterial biofilms on Aspen roots. 3D-volumes of cell structures were unstacked to deploy a panel of 2D-slices revealing the internal colony architecture. **(A)** Unstacking of a SBW25 macro-colony highlighting void spaces. One slice every 0.5 μm is shown. **(B)** Unstacking of a z-directional movie. Maximum intensity and Orthogonal projections from a reconstruction from 60 planes (left) and panel of 2D-slices revealing internal canals.

To understand the spatial organization of bacteria inside biofilm-like structures, the sequential 2D optical plans used for the 3D reconstruction were arrayed to expose the internal architecture (Figure 7, Supplementary Figure S4). SBW25 macrocolonies exhibited an internal structure with spaces devoid of bacteria that could form small channels of about 2 μm of diameter (Figure 7A). The inner structure of LP macrocolonies and DBSs displayed both small and large void spaces evocative of a network of interconnected channels (Figure 7B, Supplementary Figure S4A). Examination of the hive-like structures formed by WH6 (see Figure 3) also revealed a compact circular arrangement of bacterial cells around void spaces (Supplementary Figures S4B,C). Thus, the presence of internal void spaces appears to be a general feature of the *Pseudomonas* biofilms on Aspen roots.

**Figure 8:**
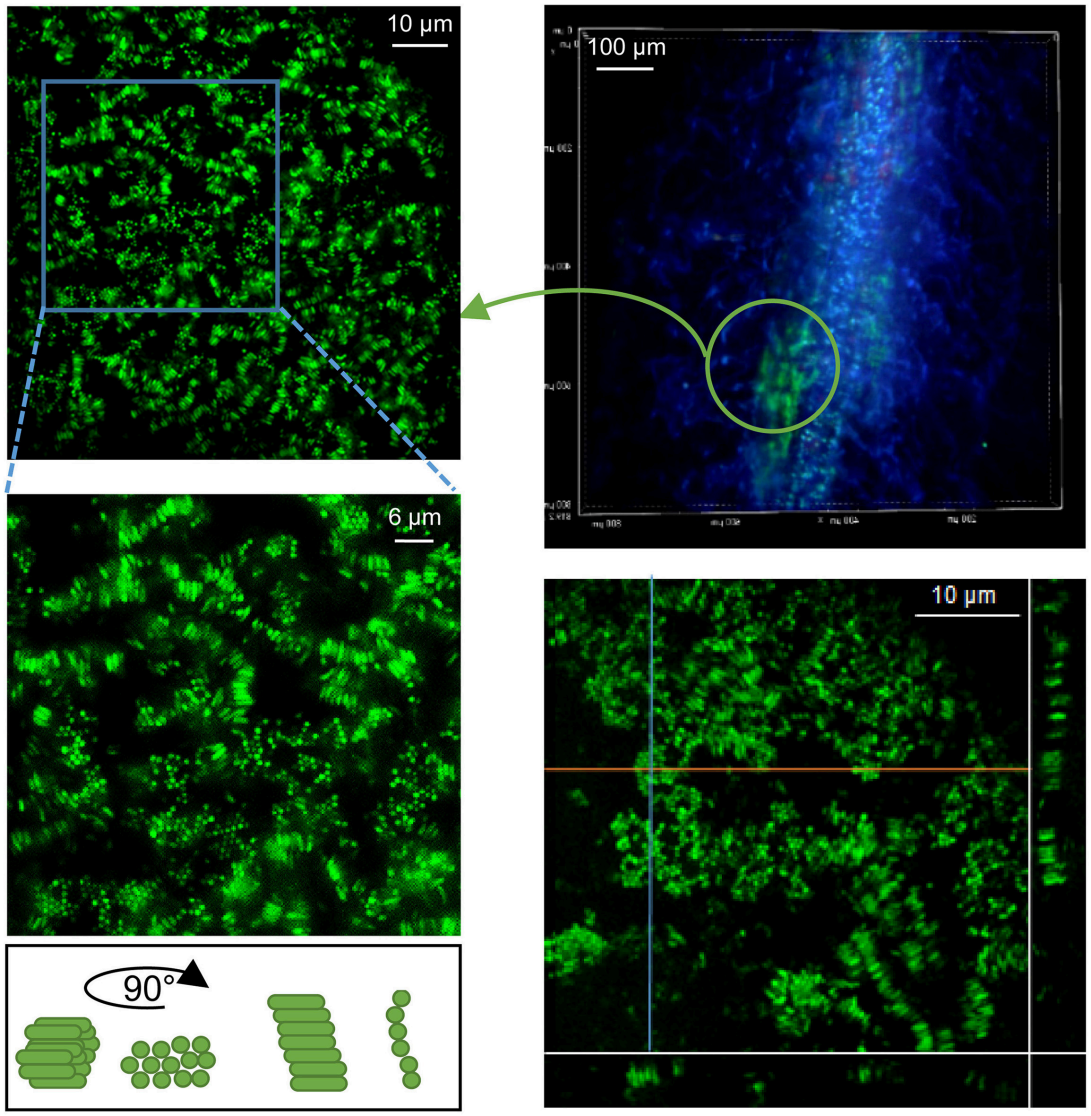
Bacteria alignment in plant-root mucilage. Close observation of *Pseudomonas* SBW25 within viscous gel-like substance present on roots (here detached from mycorrhizal roots, upper left). Epifluorescence microcopy image of SBW25 cells revealing an aligned organization. Cells appear as bundles of clustered dots or aligned rods, depending of their orientation on the mounted slide (right). Maximum Intensity Projection of a 3D-SDCM reconstruction with orthogonal slice projections (orange and blue lines) showing that the dots correspond to a subjective visualization of vertical rods.

Another type of bacterial assembly was identified at the surface of mycorrhizal roots. Densely packed SBW25 cells encased in a gelatinous substance appeared loosely associated with the surface of the fungal sheath covering the root (Figure 8). This bacterial-mucigel assembly was easily detached and observed, revealing a highly ordered bacterial arrangement. Epifluorescence and spinning disk confocal microcopy revealed a mixture of rods aligned along their long axis and honeycomb-like clustered dots (Figure 8, Supplementary Movie S2). Side projection of orthogonal slices of the 3D confocal image established that the clustered dots corresponded to a top view of vertical rods aligned along their long axis (Figure 8, Supplementary Movie S2). This nematic order suggests that biomechanical interactions between bacterial cells lead to packing along the long axis of cells within a gelatinous substratum present at mycorrhizal roots. Bacterial cells encased and aligned within the mucilage produced at the tip of mycorrhizal and non-mycorrhizal roots were also observed in seedlings cultured with SBW25 and Pf-5. These immobilized bacteria encased in a root border-cells mucilage complex can be observed forming clusters of aligned rods around the root caps (Supplementary Figure S5).

### Phenotype transition network

**Figure 9:**
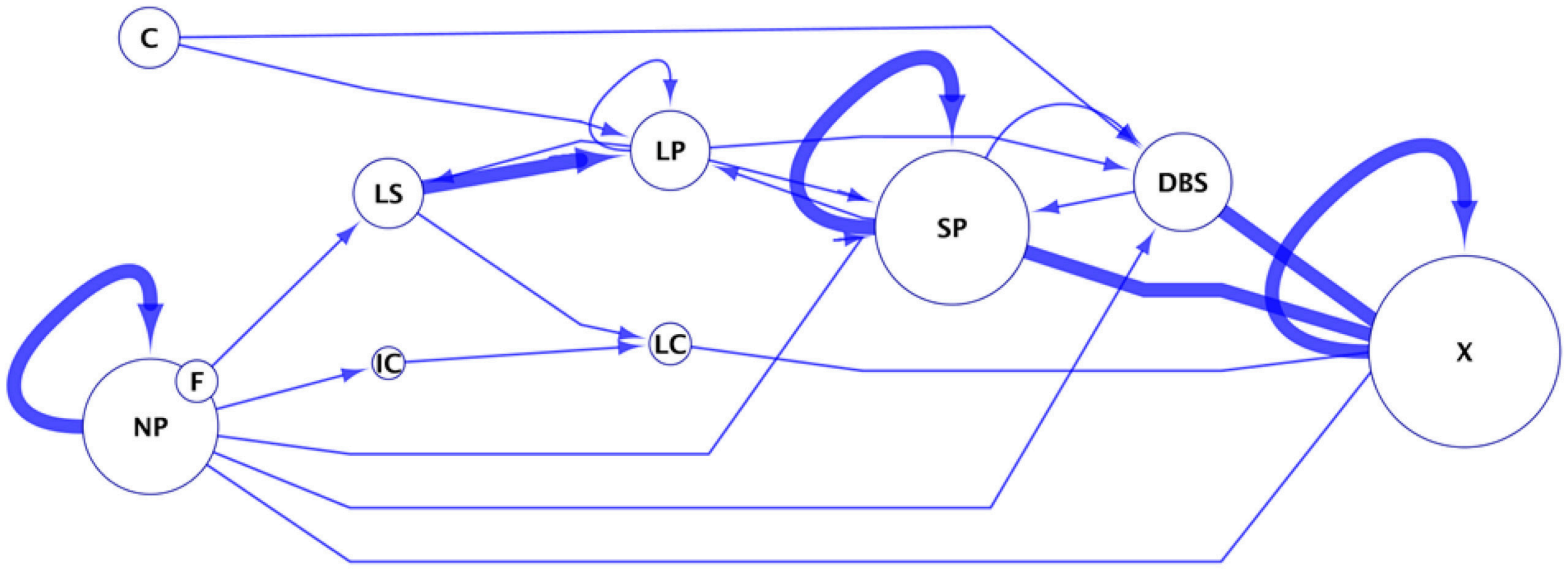
Phenotype transition network. This network was built from data summarized in Table 2. In this network, edges (lines with arrows) illustrates an observed transition of one bacterial microscopy phenotype to another. Observed phenotypes were no pattern (NP), long strips (LS), long patches (LP), short patches (SP), high density coating (C), dense biofilms structures (DBS), and filamentous cells (F). “X” represents the final state after 5 weeks, with no or too few bacteria. Edges are weighted according the frequency of that observation. Nodes are phenotypes, where the size of the node is proportional to the frequency at which that phenotype had been observed.

*Pseudomonas* strains at Aspen roots exhibit various colonization patterns, some transitioning from one to another. When considering the patterns as phenotypes (Table 2), transitions between these phenotypes (nodes) can be connected (directional arrows) over time (section Materials and Methods). We modeled the transition of bacterial patterns associated with roots as a phenotype transition network (Figure 9). The phenotypes “Non-Pattern,” “Coating,” and “Filament” are considered initial states since they were the first phenotypes observed for the 4 strains and therefore cannot derive from the other phenotypes. Only WH6 showed the phenotype “Coating” and only Pf0-1 showed the phenotype “Filament.” It is possible that SBW25 and Pf-5 either i) do not possess the “coating” and “filament” phenotypes in their repertoire or ii) that these phenotypes are not stable and are replaced by more stable phenotypes prior to the first microscopy observation. “No Pattern” can result in the highest number of possible states (6) in the network. The nodes with the highest in-degree (i.e., receiving the highest number of edges) are “SP,” “LP,” and “DBS.” These nodes form an interconnected subnetwork, in which any node in the subnetwork can be reached from any of the other nodes. The only path out of this subnetwork is the exclusion of the bacterial community from the rhizosphere, resulting in very low detected bacterial abundance. From this network, a hypothesis can be generated in which the *Pseudomonas* phenotype progresses through increasing levels of organization of cell assemblies from “LP” to “SP” to “DBS.” SBW25 is observed to most frequently possess the phenotypes “LP” and “SP” across all observations (55% for SBW25 compared to 22, 33, and 11% for Pf0-1, WH6, and Pf-5, respectively). SBW25 is the strain least likely to become extinct after forming DBS (11% for SBW25 compared with 33, 22, and 33% for Pf0-1, WH6, and Pf-5, respectively). In this model, biofilms are not necessarily an end point, but appears to be a phenotype in which the only options for the bacterial assemblies is to regain a lower level of organization.

“Dense biofilm structure” appears to be a phenotype in which the only options for the bacterial assemblies are either to regain a lower level of organization or face exclusion of bacterial cells from the community.

## Discussion

Colonization of plant roots by PGP rhizobacteria is thought to be a key component of their beneficial effects on plant development. Using the VAP system we examined the diversity of cellular assemblies formed over time at Aspen seedling roots during bipartite and tripartite interactions with *P. fluorescens* and *L. bicolor*. The four *Pseudomonas* strains used here originated from the rhizosphere of different agricultural plants and soils and were found to promote growth of Aspen seedlings, as described for many *P. fluorescens* spp. isolated form the *Populus* microbiome (Timm et al., 2015, 2016; Shinde et al., 2017). The different *Pseudomonas* strains colonized Aspen roots using different strategies. Strains SBW25 and WH6 formed highly structured biofilms at the root surface at early and late stages of colonization, respectively. Biofilm assemblies on mycorrhizal and non-mycorrhizal roots were also distinct. Thick biofilms exhibiting internal channel-like structures were observed on non-mycorrhizal root surfaces whereas at mycorrhizal roots, bacterial cells were stacked along their long axis forming a layer of cells embedded within a gel-like substance. Interestingly, SBW25 and WH6, which both formed dense biofilms at the root surface at various stages of colonization, also increased shoot biomass. In contrast, Pf0-1 and Pf-5, which did not form dense biofilms at roots, also did not increase shoot biomass. These observations suggest that biofilm formation can be associated with PGP properties in our experimental system. We found that *L. bicolor* alone promoted plant growth with no additional PGP activity resulting from post-inoculation by the *Pseudomonas* strains. The presence of *Laccaria* at seedling roots had a negative effect on bacterial persistence with all bacterial strains. One explanation could be that mycorrhization prior to exposure to the different *Pseudomonas* strains might limit their access to the rhizoplane.

It is commonly accepted that bacteria persist in their natural environments by forming biofilms in response to specific environmental cues. Many observations of bacteria on plant surfaces have revealed cellular assemblies such as aggregates, as well as micro- and macro-colonies that exhibit many characteristics of biofilms (Bogino P. C. et al., 2013). The formation of bacterial biofilms is triggered by a quorum sensing-mediated response as well as controlled by second signaling messengers (Boyd and O'Toole, 2012). Among the genetic determinants of biofilm formation in *Pseudomonas* spp., the conserved two-component (TC) regulatory system GacA/GacS is also involved in triggering life-style transition and controlling nucleotide-based secondary signaling such as the c-di-GMP signaling. The roles of the Gac system and c-di-GMP pathway in biofilm formation are described in *P. fluorescens* (Martínez-Gil et al., 2014), and our four strains encode well-conserved TCs including GacA/GacS and numerous genes encoding c-di-GMP binding proteins. However, the regulatory inputs and outputs can differ. In SBW25, the *gac* regulon comprises rhizosphere-induced genes as well as genes involved in biofilm formation such as the biosynthesis of EPS (Cheng et al., 2013). The importance of EPS in biofilm formation by *Pseudomonas* at plant roots is suggested in numerous studies but remains to be demonstrated. In other bacteria such as *Bacillus*, the ability to secrete EPS is essential for biofilm formation on Arabidopsis roots (Massalha et al., 2017). Comparison of *Pseudomonas* genomes revealed that SBW25 but not Pf0-1 nor Pf-5 have genes of the *wss* operon, which is involved in cellulose biosynthesis and important for biofilm formation (Spiers et al., 2003; Silby et al., 2009; Cheng et al., 2013). Thus, traits related to biofilm formation are differentially present in these strains, perhaps explaining differences in their ability to form biofilms at roots.

The examination of the internal architecture of the SBW25 and WH6 structure assemblies found on the rhizoplane revealed interstitial void spaces similar to channels, a characteristic of mature biofilms formed on abiotic surfaces (Wilking et al., 2013; Birjiniuk et al., 2014). *P. aeruginosa* biofilms have been described as permeated by circulatory network systems, allowing fluids to flow throughout the biofilm to exchange nutrients, oxygen, and to facilitate metabolic cooperativity (Bogino P. C. et al., 2013). Though these plant-associated dense assemblies have many hallmark of biofilms, and the mechanisms at play in SBW25 and WH6 that generate such elaborated structures remain to be investigated. The geometric shapes of some biofilms (e.g., hemisphere) suggest that mechanical forces generated by the growth of plant roots and bacterial cells shape the viscoelastic matter surrounding them. Recent studies revealed that the overall architecture of *V. cholera* biofilms was driven by the directional proliferation of the bacterial cells that align to form a hemispherical shape (Yan et al., 2016). Within confined spaces, bacteria tend to maximize their surface contact area, leading to a patterned organization (Hochbaum and Aizenberg, 2010). The spatial arrangement of SBW25 cells in a parallel honeycomb-like fashion, observed around mycorrhiza and at root caps suggests that bacteria also align within gelatinous-like substances. This particular behavior could potentially be explained by the properties of the exopolymeric gelatinous substance as in other studies bacteria were found to align with polysaccharide fibers (Lemon et al., 2017). Interestingly, mucilages are mostly composed of networks of carbohydrate fibers exhibiting a honeycomb-like ultrastructure, as observed by transmission electron microscopy (Turk et al., 2010). Bacterial alignments within mucilage-like matter could thus be considered a different form of biofilm produced at plant roots. Our observation that the *Pseudomonas* cells were embedded and vertically positioned within a mucilage substance surrounding the mycorrhizal roots also poses the question of the role of this layer in the tripartite interaction. Considering that in our *in vitro* assay bacteria were exposed to roots already extensively covered by the fungal sheath, one hypothesis is that this layer could act as a protective barrier preventing bacterial access to the plant surface. This hypothesis is in keeping with the absence of additional PGP effect of bacteria at mycorrhizal roots in the VAPs. In soil, colonization of *Populus* by *L. bicolor* was found to reach about 30% of roots (Xu et al., 2016), while in *in vitro* assays, rooted-leave cuttings were colonized at 40% after 12 weeks. Therefore, the outcome of Aspen root interactions of with *Laccaria* and *Pseudomonas* in soils will likely depend on which symbiotic microorganism is encountered first and the extent of root coverage achieved.

The formation of biofilms on rhizoplanes is not well-understood due to the dynamic nature of plant root surfaces (Rudrappa et al., 2008). Biofilm formation can be triggered at different sites by nutrient exudates and be shaped by the physico-chemical properties of the root epidermis (Ramey et al., 2004; Rudrappa et al., 2008). Theoretical models for bacterial establishment and distribution along growing roots generally take into account several aspects such as the nature of root exudate, the rate of root cell division and expansion, as well as the bacteria transport capacity. These parameters are coupled with physiological characteristics such as the carbon fluxes along the roots that can drive bacterial growth rates, behavior and dynamics (Dupuy and Silk, 2016). A recent study on colonization dynamic of Arabidopsis roots by *B. subtilis* using a microfluidic tracking device showed that *Bacillus* first colonizes the elongation zone of roots above the root tips, which correspond to the main site of exudation, and forms biofilms masses on the upper part of the roots (Massalha et al., 2017). Here, *P. fluorescens* biofilms were mostly observed on upper root parts (two-third of the root length above the tips), indicating they are matured assemblies. The colonization patterns formed overtime by the different *Pseudomonas* strains exhibited a structural progression leading to small patches (Pf0-1) or dense biofilms (SBW25, WH6). Our phenotype transition model identified a subnetwork of bacterial behaviors suggesting that biofilm formation and dispersion is a highly dynamic process, both playing a role in plant root-bacteria interaction. Although this phenotype transition model does not likely capture the full range of observable plant-bacterial interaction state dynamics, it takes into account that the progression of colonization is expected to reflect the dynamic nature of the Aspen root-bacteria interactions.

The prevalence of biofilm-forming bacteria among PGP rizhobacteria is well-recognized (Seneviratne et al., 2010; Bogino P. C. et al., 2013; Ueda and Saneoka, 2015), and raises the question about the role of biofilm formation in the PGP traits. In this regard, *Pseudomonas* SBW25 strain significantly increases Aspen seedling shoot biomass, forms stable dense biofilms at late stages of colonization, and persists longest on mycorrhizal roots. This suggests that, under our tested conditions, SBW25 possesses functional capacities that other *Pseudomonas* strains do not possess. Interestingly, the capacity of *Pseudomonas* strains to transport compounds was shown to be a strong predictor of their ecological roles in the rhizosphere (Larsen et al., 2015). This transportome capacity was previously assessed for each of the four *Pseudomonas* strains, using Predicted Relative Transmembrane Transport (PRTT) scores (Shinde et al., 2017). Unique transport capabilities can be highlighted by looking for ligands with high capacity for transport (i.e., positive PRTT scores) in SBW25 and not in the other three *Pseudomonas* strains (i.e., negative PRTT scores). Using this criterion, 11 ligand transporter functions were found to be unique to SBW25. Three of the transporter functions involve 4-aminobutanoate (GABA), arabinose, and sodium cations, which were predicted to have an effect on rootlet development in Aspen seedlings (Shinde et al., 2017). The remaining transporter functions involve multidrug export, and aminoacids, oligosaccharides and uracil transports. These candidate transporters and pathways could be directly targeted by future genetic approaches to identify the molecular mechanisms underlying *Pseudomonas* phenotype transition during colonization of plant roots.

## Conclusion

Our analysis of the spatial and temporal patterns of colonization of Aspen roots by four *Pseudomonas* strains revealed different colonization strategies. All *P. fluorescens* strains exhibited similar survival on roots but two strains (SBW25, WH6) formed dense and highly structured biofilms with internal channels whereas two strains (Pf0-1, Pf-5) did not. The ability to form biofilms correlated with PGP activities observed on a small number of seedlings in this study and on a larger number of seedlings in a previous study (Shinde et al., 2017). The role of biofilm formation on the rhizoplane in PGP remains to be better characterized. *Pseudomonas* colonization phenotypes evolved over time and the transition between these phenotypes suggest that both formation and dispersion of biofilms play a role in Aspen-bacteria interactions. Future studies of the dynamic colonization of Aspen root surfaces by microorganisms could help develop beneficial biofilm communities to improve sustainable feedstock production.

## Author contributions

All authors contributed to experimental design. SZ, PK, SS, and M-FN-G performed the biological experiments. PL performed computational analysis. M-FN-G, SS, KK, and PN contributed to critical analysis of the data. M-FN-G wrote the manuscript. All authors have read and approved the final manuscript.

### Conflict of interest statement

The authors declare that the research was conducted in the absence of any commercial or financial relationships that could be construed as a potential conflict of interest.

## Abbreviations

SDCM: Spinning Disc Confocal Microscopy
VAP: Vertical Agar Plate
PGP: Plant Growth Promotion
NM: Non-Mycorrhizal
M: Mycorrhizal
ECM: Ectomycorrhizal
MHB: Mycorrhizal Helper Bacteria
mNG: mNeongreen
mTQ: mTurquoise2
FP: fluorescent protein
LP: long patch colony
SP: short patch colony
DBS: dense biofilm-like structure
MC: macrocolony
cfu: colony forming unit
TC: Two Component system.
