# Supplementary material for: Dynamics of Aspen Roots Colonization by Pseudomonads Reveals Strain-Specific and Mycorrhizal-Specific Patterns of Biofilm Formation
Source: Front Microbiol. 2018 May 3;9:853. doi: 10.3389/fmicb.2018.00853 (PMC5943511; doi:10.3389/fmicb.2018.00853)
Supplement: Supplementary file 3 [file Presentation_1.PDF]

Supplementary Material

Dynamics of Aspen roots colonization by Pseudomonads reveals strain-specific and mycorrhizal-specific patterns of biofilm formation.

Marie-Francoise Noirot-Gros[1*], Shalaka Shinde[1], Peter E. Larsen[1], Sarah Zerbs[1], Peter J. Korajczyk[1], Kenneth M Kemner[1] and Philippe H. Noirot[1].

[1]Biosciences Division, Argonne National Laboratory, Lemont, IL, USA

Corresponding Author
mnoirot@anl.gov

Keywords: Biofilms, Plant root colonization, *Pseudomonas*, *Laccaria*, Aspen.

1. Supplementary Figures

1.1. **Supplementary Figure S1**: Chromosomal integration of the mNG fluorescent protein gene cassette in the genome of *Pseudomonas fluorescens* SBW25.
1.2. **Supplementary Figure S2**: Weekly experimental plan for studying Pseudomonas colonization patterns of non-mycorrhizal and mycorrhizal Aspen roots in vertical plates.
1.3. **Supplementary Figure S3**: Aspen root mycorrhization with Laccaria in VAPs.
1.4. **Supplementary Figure S4**: Internal architecture of biofilms structures.
1.5. **Supplementary Figure S5**: Bacterial alignments at root tips.

2. Supplementary Tables

2.1. **Supplementary Table S1**: Bacterial strains
2.2. **Supplementary Table S2**: Plasmid vectors
2.3. **Supplementary Table S3**: Primers
2.4. **Supplementary Table S4**: Genome primers

3. Supplementary Movies

3.1. **Supplementary movie S1:** P. fluorescens SBW25 on non-mycorrhizal Aspen roots at week 1 (see figure 2)

3.2. **Supplementary movie S2:** 3D-SDCM image of SBW25 cells revealing an aligned organization. Cells appear as bundles of clustered dots or aligned rods (see Figure 7)

1. Supplementary Figures

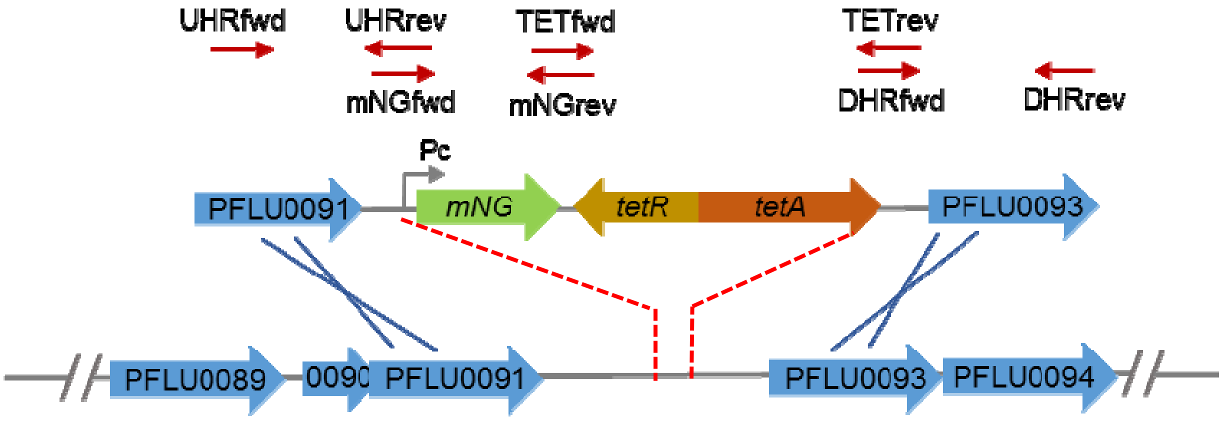

1.1. Supplementary Figure S1. Chromosomal integration of the mNeonGreen fluorescent protein cassette in the genome of *Pseudomonas fluorescens* SBW25. The example shown for SBW25 genome is representative of the strategy used for all *Pseudomonas* strains in this study. The insertion sites for the cassette expressing fluorescent protein and antibiotic resistance genes, were selected to be intergenic regions expected to be neutral genomic locations. To generate a mNG-labeled SBW25 strain, a linear DNA fragment containing the flanking PFLU0091 and PFLU0093 genes, the fluorescent protein gene (mNG), the tetracycline efflux pump *tetA* and tetracycline-responsive regulator *tetR* genes was constructed using PCR and assembly cloning (see Table S1). The resulting 5122 bp fragment was then transformed into *P. fluorescens* SBW25 cells harboring a plasmid expressing the *RecET* recombinases under the control of an arabinose-inducible promoter. Transformants were selected, verified for mNG expression and for by expected genome structure colony PCR, and finally cured of the recombinase plasmid. The resulting SBW25 strain constitutively expressed mNG from a stable chromosomally-integrated cassette. A similar strategy was used to insert the mNG within the PFL_0094-0095 region in *Pf-5*, the Pfl01_0042-0043 region in Pf0-1 and the PFWH6_0090-0092 region in WH6. In the Pf0-1 strain the Pfl01_0042-0043 region reads in the opposite direction relative to the rest of the chromosome so the mNG-gene was inserted to the same orientation of the surrounding genes. Pf-5 was labeled was labelled by insertion of a mTurquoise fluorescent protein expressed from the constitutive Pc promoter and associated with a gentamycin resistance gene (Table S1).

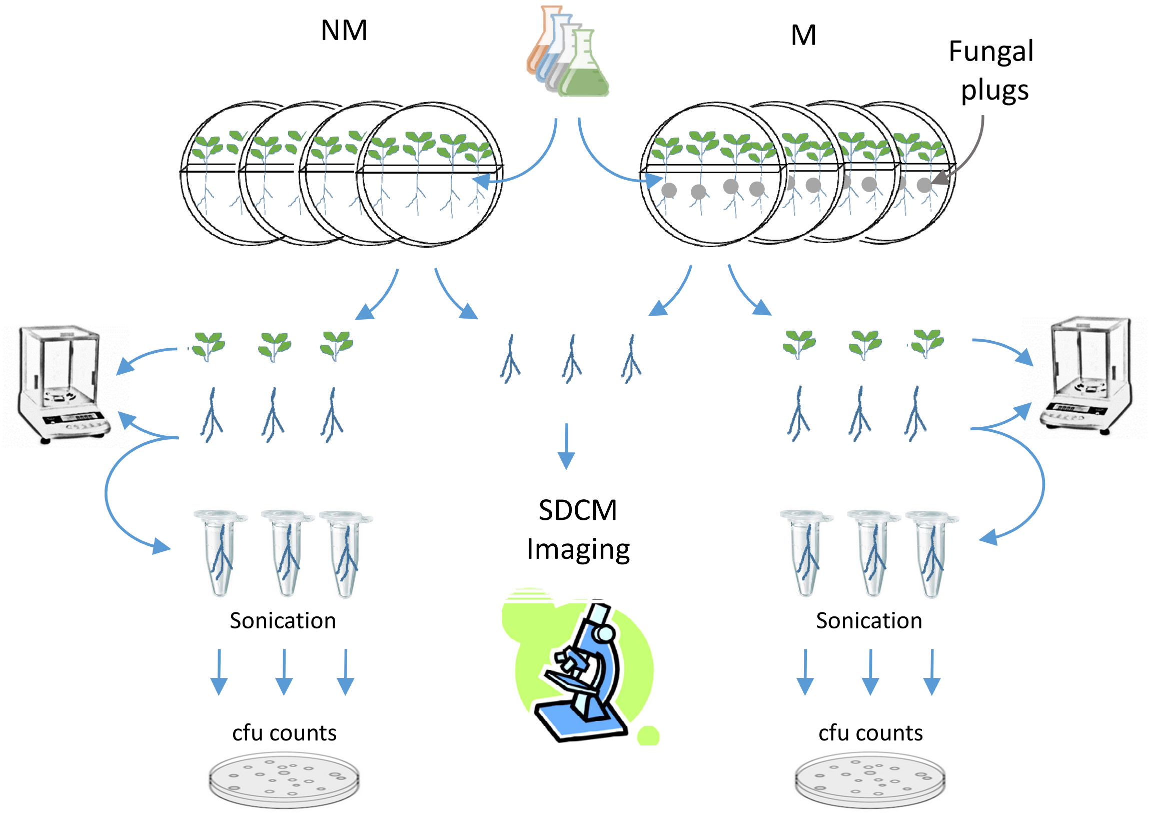

1.2. Supplementary Figure S2. Weekly experimental plan for studying *Pseudomonas* colonization patterns of non-mycorrhizal and mycorrhizal Aspen roots in vertical plates. Aspen seedlings were transferred one week after germination onto vertical agar plates either untreated or previously colonized by Laccaria (see Materiel and Methods). After one week, the roots of seedlings were inoculated with the different FP-labeled *Pseudomonas* strains one week after transfer to the vertical plate by applying 10µl of bacteria suspension ($4x10^9$). Three seedlings were sampled each week and treated separately. For each seedling, whole shoots and roots systems were separated for weighting. Roots were then immersed in PBS and bacteria were detached by sonication (40 Htz for 5 minutes in a water filled sonication bath). Detached bacteria were enumerated by plating on LB plate containing the appropriated antibiotic (Tetracycline 10µg/ml for SBW25, WH6 and Pf0-1, and Gentamycin 10µg/ml for Pf5). Additionally, 2 to 3 root seedlings were taken for the observation of the bacterial colonization pattern by Spinning Disk Confocal Microscopy.

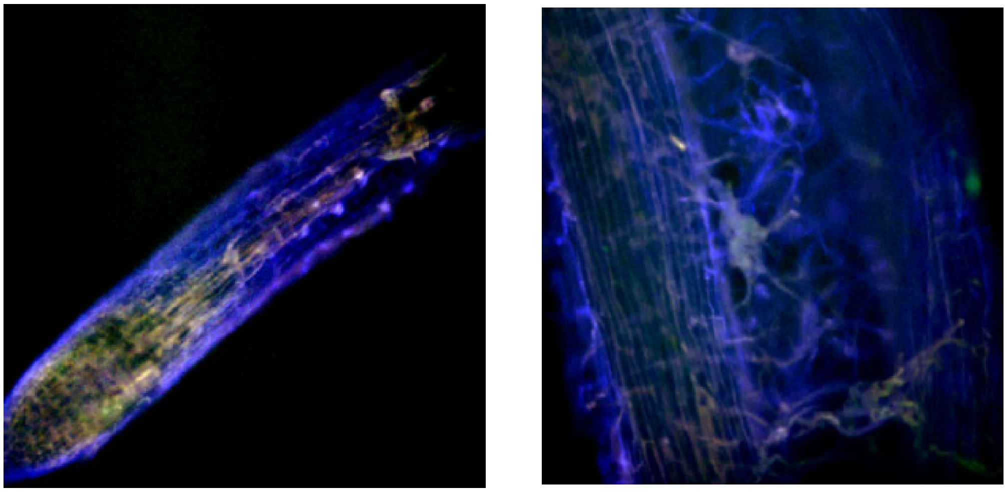

1.3. Supplementary Figure S3.

Aspen root mycorrhization with Laccaria in VAPs. Epifluorescence microcopy of Aspen roots one week after transfer to VAP. Calcofluor White dye staining reveals the fungal sheath around the roots.

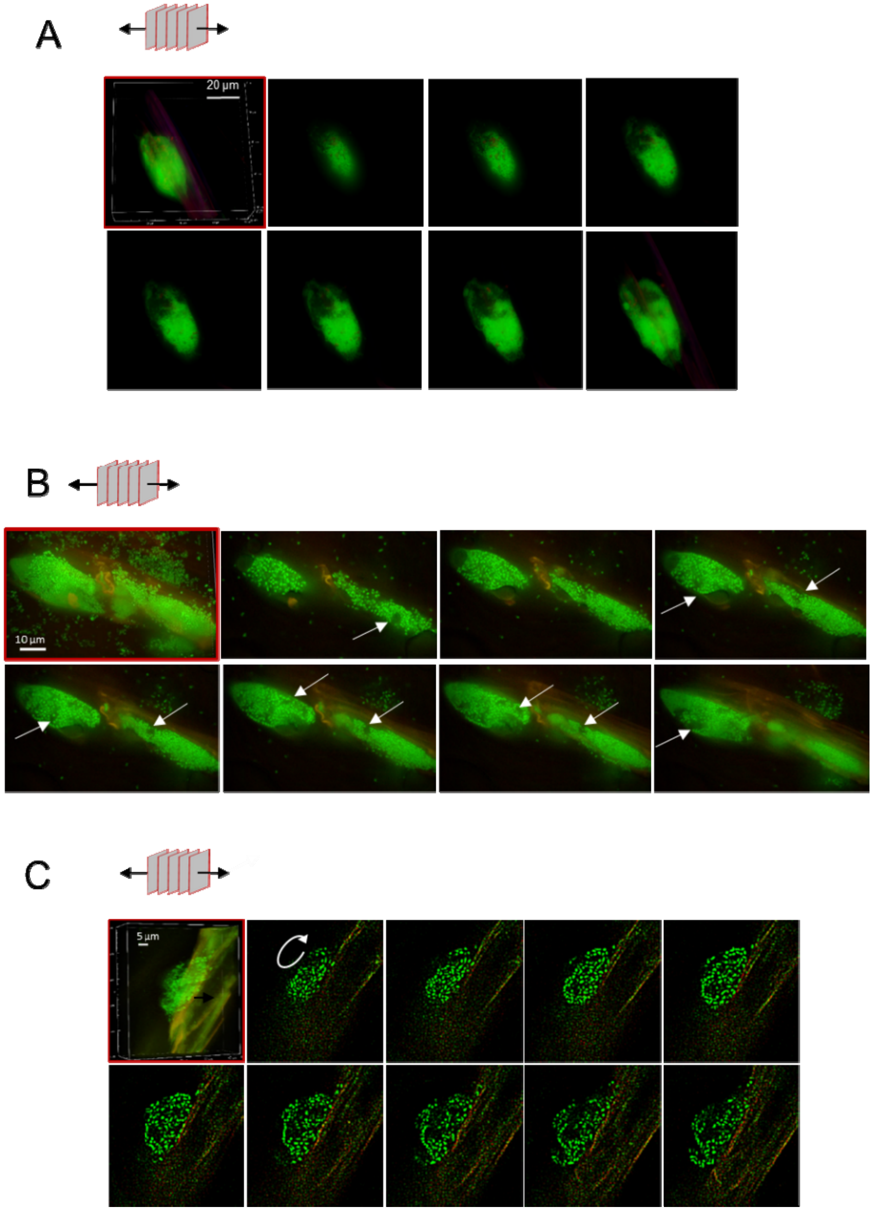

1.4. Supplementary Figure S4. Internal architecture of biofilms structures. 2D images used for 3D reconstruction of biofilm structures were arrayed in sequence to expose internal structures. (A) SBW25 DBS displays void spaces. One 2D plan every 1 µm is shown. (B) WH6 biofilms internal structure. A hive-like biofilm (see Figure 3) also exhibits internal void spaces (arrows). (C) WH6 hemispheric structure. A Laplacian convolution treatment was applied to highlight the quasi-circular arrangement of bacterial cells around void spaces.

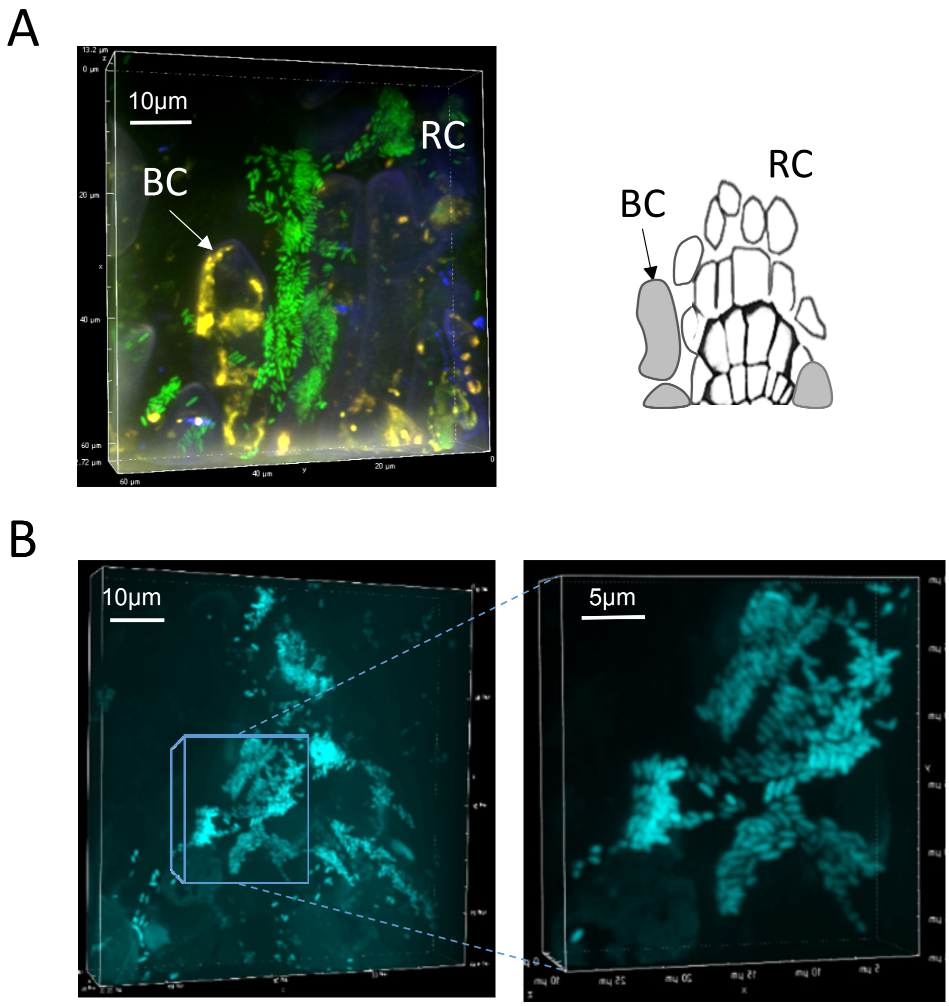

1.5. Supplementary Figure S5. Bacterial alignments at root tips. (A) 3D-volumes displaying the surface arrangements of SBW25 cells at non-mycorrhizal root tips. Cells aligned within a mucilage substance around the border cells and root cap. Right shows a schematic of root tip with border cells in the same orientation than the root tips. B) 3D-volumes displaying the surface arrangements of Pf-5 with a blow-up (right) showing vertically aligned bacteria.

2. Supplementary tables:

2.1. Supplementary Table S1:

A- Bacterial Strains		
**Species**	**Name**	**Description**
E.coli	NEB Turbo	New England Biolabs
P.protegens PF-5	wildtype	ATCC
P. protegens PF-5	PFL_0094:mTurquoise2-GentR:0095	Pc_mTurquoise2; Gentamycin-selective; this work
P. fluorescens Pf0-1	wildtype	from M.Silby (University of Massachusetts, Dartmouth)
P. fluorescens Pf0-1	PFL01_0042:TetAR-mNeongreen:0043	Pc_mNeongreen; Tetracycline-selective; this work
P.fluorescens SBW25	wildtype	from G. Preston (University of Oxford)
P.fluorescens SBW25	PFLU0091:mNeongreen-TetAR:0093	Pc_mNeongreen; Tetracycline-selective; this work
P. fluorescens WH6	wildtype	from D. Armstrong and M. Azevedo (Oregon State University)
P.fluorescens WH6	PFWH60090:mNeongreen-TetAR:0092	Pc_mNeongreen; Tetracycline-selective; this work

2.2. Supplementary Table S2:

Vectors		
**Name**	**Source**	**Description**
pUC19	New England Biolabs	Standard *E. coli* cloning vector; Ampicillin-selective
pME6031-Pc-mNeongreen	R. Wilton (ANL, Lemont, IL)	Rhizosphere-stable; Shuttle vector pME6031; TetR
pME6031-PsbA-mTurquoise2	R. Wilton (ANL, Lemont, IL)	Rhizosphere-stable; Shuttle vector pME6031; TetR
pSEVA614	V. di Lorenzo (CNB, Madrid, Spain)	Modular vector; Gentamycin selective
pUC19-Pc-mNeongreen	this work	pUC derivative, replicative in P. fluorescens, AmpR and TetR
pUC19-Pc-mTurquoise	this work	pUC derivative, replicative in P. fluorescens, AmpR and GnR
pUCP24-PsyrRecET	B. Swingle (USDA-ARS, Ithaca, NY)	Recombineering vector expressing RecET recombinase genes derived from *P. syringea*; GnR
pENG23-PFLURecETγ	Korajczyk et al. (in preparation)	Recombineering vector for arabinose-inducible expression of RecET recombinase genes derived from *P. fluorescens* SBW25; KnR

2.3. Supplementary Table S3:

Construction Primers		
**Name**	**Sequence**	**Target**
PmeI-mNGc-Tetshort	GGATCAGTGAGGGTTT-AAACTGGCTCTGCTGTAGTGAG	TetAR
pUCb-tetshort	TGAATTCGAGCTCGGTACCC-TAGAGCGGCCTATCGTTTC	TetAR
PmeI fwd-GentR	GGATCAGTGAGGGTTT-AAACGGACCGTTGTCCAATTTAC	GentR
pUC19b rev-GentR	TGAATTCGAGCTCGGTACCC-CGGGCGTTTTTTATTGGTG	GentR
pUC19a-Pc-mNGshort	GTCGACTCTAGAGGATCCCC-GACGCGTTGCCGATAC	Pc-mNeongreen
mNGc-PmeIshort	GTTTAAACCCTCACTGATCCGC	Pc-mNeongreen
pUC19-Pc fwd-mTurquoise	ACTTTAAGAAGGAGATATACATATGGCTAGCAAGGGCG	promoterless mTurquoise2
PmeI rev-mTurquoise	GTTTAAACCCTCACTGATCCTCACTTGTACAGCTCGTCCATG	promoterless mTurquoise2
pUC19-Pc rev	CATATGTATATCTCCTTCTTAAAGTTAAAC	pUC19-Pc promoter
pUC19b fwd	GGGTACCGAGCTCGAATTC	pUC19-Pc promoter
pUC19a-Pc-mNGshort	GTCGACTCTAGAGGATCCCC-GACGCGTTGCCGATAC	Pc-mTurquoise2
mNGc-PmeIshort	GTTTAAACCCTCACTGATCCGC	Pc-mTurquoise2

2.4. Supplementary Table S4:

Genome Primers				
**Strain**	**Targeted Region**	**Primer ID**	**UHRfwd seq**	**5' genome location**
PF-5	PFL_0094-0095	PFL_0094 fwd	AGCTGGGCAATCACATTC	94194
Pfl0-1	Pfl01_0042-0043	Pfl01_0042 fwd	GAAACCGAAGTTGGCCAC	46664
SBW25	PFLU0091-0093	0091-0093 forward	GATCATCGCCATTGTGGCTG	91627
WH6	PFWH6_0090-0092	PFWH6_0090 fwd	CATGTTGAGTTGGGCAATC	draft
**Strain**	**Targeted Region**	**Primer ID**	**UHRrev seq**	**5' genome location**
PF-5	PFL_0094-0095	PFL_0094-pUCa rev	GGGGATCCTCTAGAGTCGAC-GCAATTCGCACAAAGTTTC	95195
Pfl0-1	Pfl01_0042-0043	Pfl01_0042-pUCb fwd	GGGTACCGAGCTCGAATTCA-TTTAGTTTCCGTACGTAGGC	47695
SBW25	PFLU0091-0093	0091-0093-pUCa	GGGGATCCTCTAGAGTCGAC-CGCCAGATATGTCCTATAGCG	92643
WH6	PFWH6_0090-0092	PFWH6_0090-pUCa rev	GGGGATCCTCTAGAGTCGAC-GAAATGGCCGATAGAGGC	draft

Strain	Targeted Region	Primer ID	DHRfwd seq	5' genome location
PF-5	PFL_0094-0095	PFL_0094-pUCb fwd	GGGTACCGAGCTCGAATTCA-TTGCGCAGCGTTTCCG	95192
Pfl0-1	Pfl01_0042-0043	Pfl01_0042-pUCa rev	GGGGATCCTCTAGAGTCGAC-AAATGTCCGACTTGGTGG	47693
SBW25	PFLU0091-0093	0091-0093-pUCb	GGGTACCGAGCTCGAATTCA-GTCAGGTCGCGGATTCC	92643
WH6	PFWH6_0090-0092	PFWH6_0090-pUCb fwd v2	GGGTACCGAGCTCGAATTCA-GCCGAATTCCCTAGCGTTTGC	draft
**Strain**	**Targeted Region**	**Primer ID**	**DHRrev seq**	**5' genome location**
PF-5	PFL_0094-0095	PFL_0094 rev	GTGCAAATACGCCGATAG	96204
Pfl0-1	Pfl01_0042-0043	Pfl01_0042 rev	CCGGCAACATCCCTAAATAG	48698
SBW25	PFLU0091-0093	0091-0093 reverse	GGCGTACTTCATGATCATGTG	93617
WH6	PFWH6_0090-0092	PFWH6_0090 rev v2	GGGTGACTTTGAACATGCTCTC	draft

3. Supplementary Movies

3.1. **Supplementary Movie S1:** P. fluorescens SBW25 on non-mycorrhizal Aspen roots at week 1 (see figure 2)

3.2. **Supplementary Movie S2:** 3D-SDCM image of SBW25 cells revealing an aligned organization. Cells appear as bundles of clustered dots or aligned rods (see Figure 7)
